# The Protease SplB of Staphylococcus aureus Targets Host Complement Components and Inhibits Complement-Mediated Bacterial Opsonophagocytosis

**DOI:** 10.1128/JB.00184-21

**Published:** 2022-01-18

**Authors:** Prasad Dasari, Maria Nordengrün, Cláudia Vilhena, Leif Steil, Goran Abdurrahman, Kristin Surmann, Vishnu Dhople, Julia Lahrberg, Claus Bachert, Christine Skerka, Uwe Völker, Barbara M. Bröker, Peter F. Zipfel

**Affiliations:** a Department of Infection Biology, Leibniz Institute for Natural Product Research and Infection Biology, Hans Knöll Institute, Jena, Germany; b Institute of Immunology, University Medicine Greifswald, Greifswald, Germany; c Interfaculty Institute of Genetics and Functional Genomics, University Medicine Greifswald, Greifswald, Germany; d Upper Airways Research Laboratory, Department of Otorhinolaryngology, Ghent University, Ghent, Belgium; e Institute of Microbiology, Friedrich Schiller University, Jena, Germany; University of Illinois at Chicago

**Keywords:** complement, immune evasion, innate immunity

## Abstract

Staphylococcus aureus is an opportunistic pathogen that can cause life-threatening infections, particularly in immunocompromised individuals. The high-level virulence of S. aureus largely relies on its diverse and variable collection of virulence factors and immune evasion proteins, including the six serine protease-like proteins SplA to SplF. Spl proteins are expressed by most clinical isolates of S. aureus, but little is known about the molecular mechanisms by which these proteins modify the host’s immune response for the benefit of the bacteria. Here, we identify SplB as a protease that inactivates central human complement proteins, i.e., C3, C4, and the activation fragments C3b and C4b, by preferentially cleaving their α-chains. SplB maintained its proteolytic activity in human serum, degrading C3 and C4. SplB further cleaved the components of the terminal complement pathway, C5, C6, C7, C8, and C9. In contrast, the important soluble human complement regulators factor H and C4b-binding protein (C4BP), as well as C1q, were left intact. Thereby, SplB reduced C3b-mediated opsonophagocytosis by human neutrophils as well as C5b-9 deposition on the bacterial surface. In conclusion, we identified the first physiological substrates of the S. aureus extracellular protease SplB. This enzyme inhibits all three complement pathways and blocks opsonophagocytosis. Thus, SplB can be considered a novel staphylococcal complement evasion protein.

**IMPORTANCE** The success of bacterial pathogens in immunocompetent humans depends on the control and inactivation of host immunity. S. aureus, like many other pathogens, efficiently blocks host complement attack early in infection. Aiming to understand the role of the S. aureus-encoded orphan proteases of the Spl operon, we asked whether these proteins play a role in immune escape. We found that SplB inhibits all three complement activation pathways as well as the lytic terminal complement pathway. This blocks the opsonophagocytosis of the bacteria by neutrophils. We also clarified the molecular mechanisms: SplB cleaves the human complement proteins C3, C4, C5, C6, C7, C8, and C9 as well as factor B but not the complement inhibitors factor H and C4BP. Thus, we identify the first physiological substrates of the extracellular protease SplB of S. aureus and characterize SplB as a novel staphylococcal complement evasion protein.

## INTRODUCTION

The opportunistic human pathogen Staphylococcus aureus colonizes around 20% of the world’s population persistently and the remainder of the population intermittently, usually without clinical symptoms (1–3). However, these bacteria can also cause community-associated and nosocomial infections. S. aureus is a common cause of superficial skin and soft tissue infections and, especially in immunocompromised individuals, can induce life-threatening systemic infections such as pneumonia, sepsis, and osteomyelitis. Antibiotic resistance further complicates clinical courses in many cases (4, 5). Finally, S. aureus is associated with allergies, in particular with atopic dermatitis and allergic airway diseases (6, 7). During its multifaceted interaction with the human host, S. aureus can rely on a broad panel of virulence and immune evasion factors, including secreted toxins and extracellular enzymes (8–12).

Human complement is a humoral immune surveillance system that networks with innate and adaptive immune responses (13). The complement system protects the host from invading microbes by activating a self-amplifying proteolytic cascade that results in the rapid elimination of invading microbes (14).

Complement activation can occur via the alternative, classical, and lectin pathways. The alternative complement pathway forms the first line of defense against infectious microbes and generates an amplification loop in classical and lectin pathway activation. All three pathways result in the formation of C3 convertase. Surface-assembled C3 convertases (C3bBb and C4b2a) cleave the central complement protein C3, generating C3a, a potent antimicrobial and anaphylatoxin protein, and the opsonic fragment C3b, which covalently attaches to the surface of bacteria and marks these targets for phagocytosis. When C3b attaches to an existing C3 convertase, e.g., on the bacterial surface, C5 convertases (C4b3b2a and C3bBbC3b) are formed, which cleave C5 into the potent anaphylatoxin C5a and the reactive C5b fragment that also attaches to bacterial surfaces. C5b can subsequently recruit C6, C7, C8, and multiple copies of C9 to form the terminal complement complex, also termed the membrane attack complex, which lyses the target cell. To protect host cells from damage by activated complement, the activation of complement is tightly regulated by soluble and membrane-bound host complement regulators (13). The plasma C4b-binding protein (C4BP) blocks C3 convertases of the classical and lectin pathways by factor I-mediated cleavage of C4b. Factor H dissociates the C3 convertase of the alternative pathway and has cofactor activity for factor I-assisted inactivation of C3b. Furthermore, membrane complement regulators, such as CD46, complement receptor 1 (CR1), and CD55, dissociate C3 convertase, while CD59 blocks the integration of the terminal complement complex into the target membrane.

Underscoring the importance of complement in infection control, many microbial pathogens, including Gram-negative and Gram-positive bacteria, as well as fungi and multicellular parasites, have evolved related strategies to interfere with and block toxic complement effector functions (14, 61). These include (i) the production of a capsule to avoid complement recognition and to shield the surface, (ii) the recruitment of host complement regulators to the bacterial surface, (iii) the secretion of proteases that directly inactivate complement proteins by degradation, and (iv) the expression of surface proteins that bind to the Fc region of immunoglobulin to block the classical complement pathway (15–17).

S. aureus commands numerous means to evade host complement-mediated damage (8, 10). On its surface, this pathogen expresses protein A (SpA) and the secreted staphylococcal binder of IgG (Sbi), which bind to the Fc region of IgG and block C1q-dependent complement activation via the classical pathway (15–17, 18, 59). Furthermore, S. aureus staphylokinase cleaves the proenzyme plasminogen into active plasmin, which in turn inactivates C3 and C3b, and also cleaves IgG and extracellular matrix components to inhibit the classical complement pathway (19). Moreover, S. aureus expresses proteins that recruit host complement inhibitors to its surface to mediate complement evasion. S. aureus surface-located serine-aspartate repeat-containing protein E (SdrE) recruits both factor H and C4BP, while the bone sialoprotein-binding protein (Bbp) binds C4BP and limits opsonophagocytosis and bacterial killing. Extracellular fibrinogen-binding protein (Efb), the Efb-homologous protein (Ehp), as well as the extracellular complement-binding protein (Ecb) directly bind C3 and C3d and block C3 convertase formation (20–22). The staphylococcal complement inhibitor (SCIN) binds to and stabilizes C3 convertases, thereby reducing the C3 convertase activity of all three pathways (23). Moreover, aureolysin (Atl), a metalloprotease released by S. aureus, directly cleaves the central complement molecule C3 in serum, where the resulting fragments are then degraded by host complement inhibitors (24). As a result, less complement is deposited on the bacterial surface, and opsonophagocytosis by neutrophils is reduced.

In addition to aureolysin, S. aureus can release two cysteine proteases, staphopain A (ScpA) and staphopain B (SspB), and nine serine proteases, among them staphylococcal serine protease A (SspA or V8 protease) as well as exfoliative toxins A and B (Eta and Etb). Their pathophysiological functions are well documented and are summarized in an excellent recent review (11). In contrast, the physiological roles of the six other staphylococcal serine proteases, termed staphylococcal serine protease-like proteins SplA to SplF, have remained a mystery. They were discovered 2 decades ago and are all encoded in the *spl* operon of S. aureus (25, 29). The deletion of the *spl* operon influenced the surface expression of numerous bacterial proteins, including many virulence factors. While this did not change bacterial survival in a rabbit pneumonia model, the six Spl proteins appeared to favor bacterial dissemination in the lungs (26). The human immune response to Spl proteins is biased toward the type 2 immune effector module characterized by the release of interleukin-4 (IL-4), IL-5, and IL-13 as well as by specific IgE and IgG4 (27). This is unusual for S. aureus antigens, which tend to elicit interferon gamma (IFN-γ)- and IL-17-dominated responses typical of the type 1 and 3 effector modules (27). Moreover, SplD can elicit strong allergic airway inflammation in mice, mediated by IL-33 (27, 28).

Spl proteases share a limited sequence identity of around 40 to 70%, except for SplD and SplF, which are 95% identical (25, 29). In bacterial cell cultures, Spl proteins are released during the early stationary growth phase (29). The molecular structure and function as well as the cleavage motifs of SplA, SplB, SplD, and SplE have been systematically elucidated (30–36). However, the physiological substrates of Spl proteins are still largely elusive. The only substrate identified thus far is mucin 16, which is degraded by SplA (26).

Here, we show that SplB cleaves and inactivates several human complement proteins and blocks opsonophagocytosis by human neutrophils as well as the formation of the terminal complement complex.

## RESULTS

### Composition of the *spl* operon in nasal S. aureus isolates.

**FIG 1:**
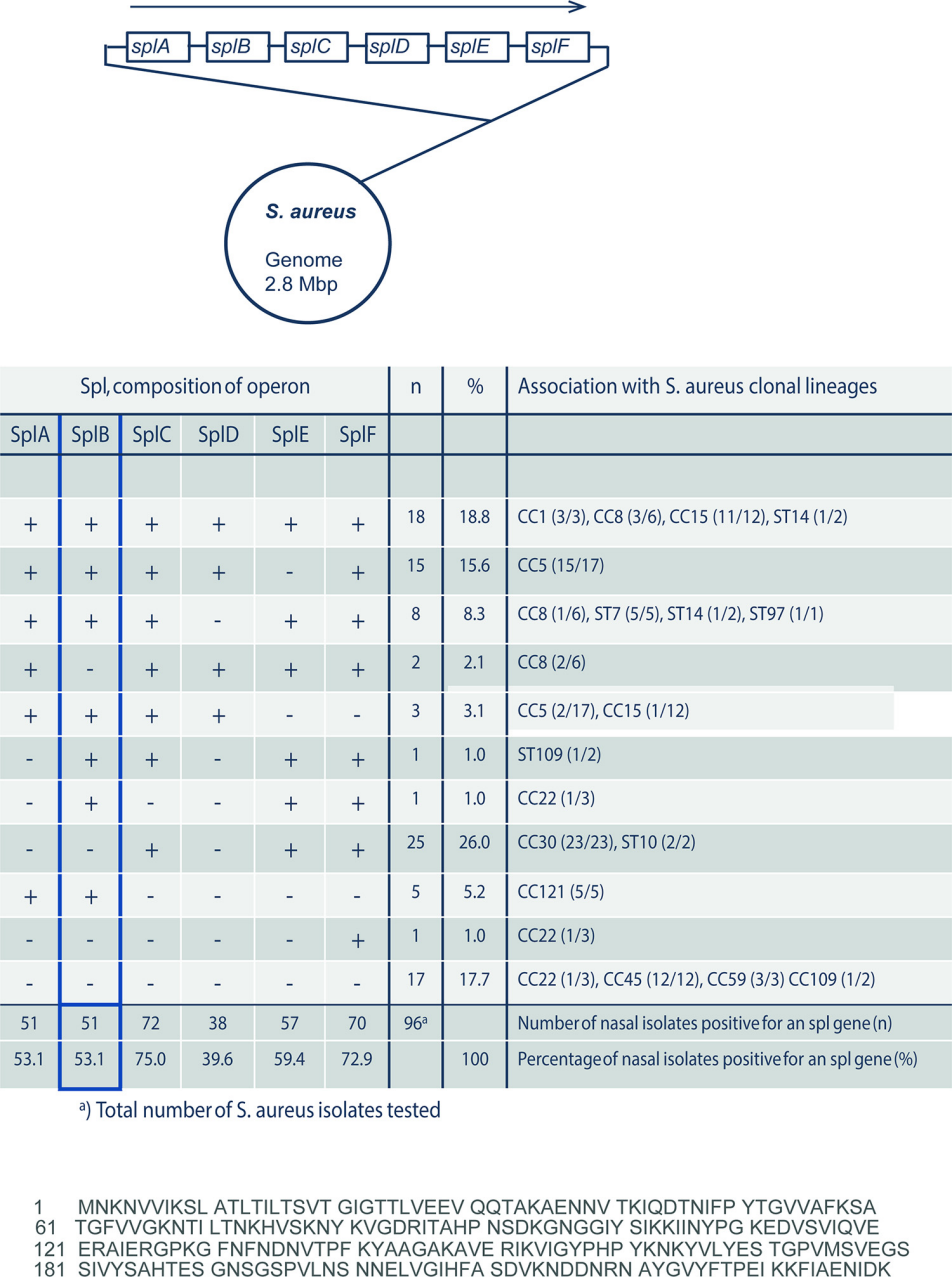
The *spl* operon of S. aureus. (A) Schematic representation of the complete *spl* operon of S. aureus. (B) Composition of the *spl* operon in human nasal S. aureus isolates. (C) SplB sequence.

The genomes of many S. aureus isolates contain *spl* operons that can include up to six genes. As the gene products include related protease motifs, the proteins are termed serine protease-like proteins (SplA to SplF) (Fig. 1A) (29). Given the variable compositions of the *spl* operon in S. aureus isolates and the sequence heterogeneity between the genes and proteins, we were first interested in evaluating the distribution and composition of the operon in S. aureus clinical isolates.

To this end, we assessed the presence and composition of the *spl* operon in 96 nasal S. aureus strains derived from healthy carriers. Five dominant clonal clusters (CCs) (CC30, CC5, CC15, CC45, and CC8) accounted for 73% of the strains. Most isolates contained an *spl* operon (*n *= 79; 82.3%). Seventeen isolates lacked this operon; of these, 12 strains belonged to the clonal lineage CC45. The composition of the *spl* operon was diverse and closely linked to the genetic background of the bacteria. Eighteen isolates (18.8%) had all six *spl* genes, and 61 isolates (63.5%) had between one and five *spl* genes. As a result, single *spl* genes varied in frequency from 39.6% (*splD*) to 75.0% (*splC*) in clinical S. aureus strains. SplB was present in approximately half of the clinical isolates (53.1%) (Fig. 1B).

### Expression and purification of SplA to SplF.

To address the role of individual Spl proteins, the genes encoding SplA, SplB, SplC, SplD, SplE, and SplF of S. aureus USA300 were cloned into Bacillus subtilis as tag-free recombinant variants lacking the signal sequence. The proteins were expressed and purified to homogeneity from bacterial supernatants (see Fig. S1 in the supplemental material).

### SplB directly cleaves and inactivates the central complement protein C3.

**FIG 2:**
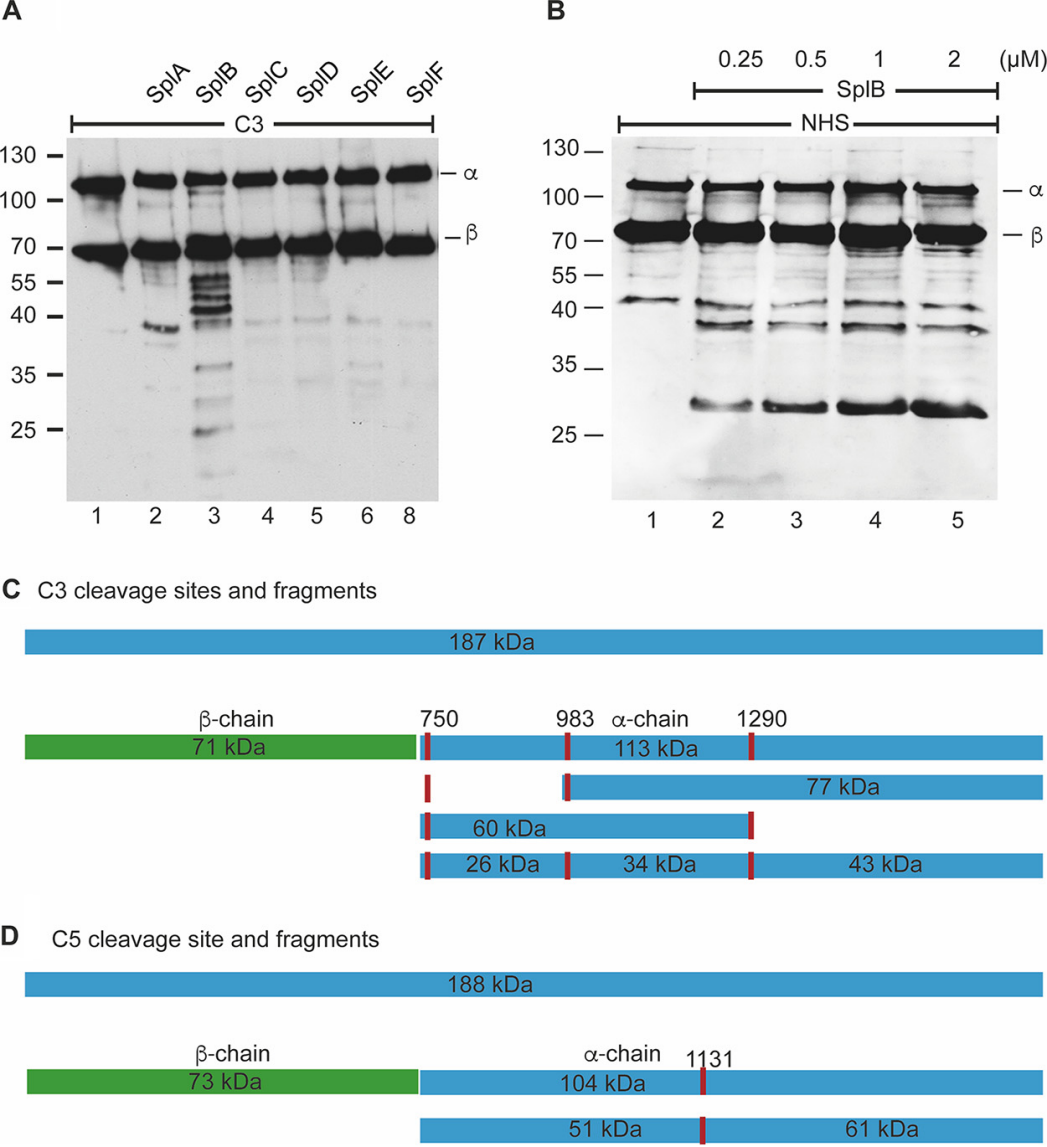
SplB cleaves the complement proteins C3 and C5. (A) SplA to -F (1 μM) were incubated with 1 μg of C3 for 2 h at 37°C, and after separation by SDS-PAGE, the cleavage patterns were visualized by Western blotting with an anti-human C3 antiserum. Only SplB showed prominent proteolytic activity toward C3 and digested its α-chain into several distinct fragments. (B) Increasing concentrations of SplB were incubated with 0.5% NHS as the source of complement, and the reaction mixture was separated by SDS-PAGE. Western blotting with a goat anti-human C3 antiserum revealed concentration-dependent cleavage of C3 by SplB. (C) Schematic representation of the cleavage sites (P1 [red bars]) targeted by SplB in the complement protein C3 and the resulting fragment sizes. The cleavage sites were determined by mass spectrometric identification of N termini. (D) Schematic representation of the cleavage site (P1) targeted by SplB in the complement protein C5 and the resulting fragment sizes.

We hypothesized a role in immune evasion and therefore asked whether Spl proteins can cleave and inactivate the central human complement protein C3. Each Spl variant was incubated with human C3 for 2 h at 37°C, and the C3 cleavage fragments were analyzed by Western blotting. SplB cleaved C3, as visualized by the reduced intensity of the α-chain and the appearance of new bands with lower mobility (Fig. 2A). SplB mainly targeted the α-chain of C3, while the β-chain remained intact (Fig. 2A; Fig. S2B and C). When incubated with culture supernatants from a B. subtilis expression strain lacking an *spl* expression vector, C3 remained intact, which excludes the possibility of cleavage by contaminating B. subtilis proteases (Fig. S2A). SplB-mediated cleavage was concentration and time dependent (Fig. S2B and C).

SplA, SplC, SplD, SplE, and SplF lacked such prominent C3 cleavage activity; however, in the presence of these proteins, new bands also appeared but at a lower intensity. This shows the preference of SplB for human C3 (Fig. 2A).

### SplB cleaves C3 in human serum.

To counteract host complement attack during infection, bacterial proteases must resist protease inhibitors that are present in human plasma. Therefore, we evaluated C3 cleavage by SplB in normal human serum (NHS), which also served as the source of C3. Immunoblot analysis revealed that SplB also processed C3 in human serum. The α-chain was cleaved, and fragments with mobilities of approximately 70, 38, and 30 kDa appeared. This cleavage was concentration dependent (Fig. 2B). Thus, SplB is active in human serum, where C3 cleavage seems more restricted than in buffer.

### N-terminal sequences of C3 cleavage products generated by SplB.

To localize cleavage sites in the C3 α-chain, we determined the N-terminal sequences of the SplB-generated C3 fragments by mass spectrometry. SplB cleaved the α-chain of C3 after amino acids 750, 983, and 1290 (Table 1 and Fig. 2C). The calculated masses of the fragments were in good agreement with their mobility upon SDS-PAGE separation. SplB preferentially cleaved after glutamine residues, as two cleavage events occurred after glutamine, and one event occurred after asparagine (position P1) (Table 1). Position P2 was variable, and 2 of the 3 amino acids at the P1′ position were acidic.

**TABLE 1:** SplB and aureolysin cleavage sites in the complement proteins C3, C4, and C5

Peptide^*a*^	Cleavage motif							Cleavage efficiency (%)^*b*^		
	P5	P4	P3	P2	P1	P1′	P2′	SplB	Aureolysin	SplB + aureolysin
C3										
CS_A 751–764^*c*^	L	A	R	S	N	L	D	>10	>100^*d*^	>100^*d*^
CS_7 984–999	R	I	L	L	Q	G	T	>10	0^*d*^	0^*d*^
CS_5 1291–1303	A	P	D	H	Q	E	L	>10	0^*d*^	0^*d*^

C4										
CS_1 763–775	L	E	I	L	Q	E	E	>100	ND	ND
CS_5 1113–1126	L	S	Q	Q	Q	A	D	>10	ND	ND

C5										
CS_1 1131–1138	P	I	K	L	Q	G	L	>100^*d*^	ND	ND

a Peptides generated by enzymatic cleavage.

b The cleavage efficiency is expressed as the ratio between a peptide’s abundances in the presence and absence of SplB. Only results with a ratio of >10 are included. ND, not determined.

c Amino acid positions of the new N-terminal peptides generated by enzymatic fragmentation. The first (N-terminal) amino acid position of the generated peptide corresponds to position P1′ of the cleavage motif.

d Result of one experiment. In all other cases, two experiments were performed, with similar results.

### SplB also inactivates C3 activation fragments.

SplB cleaved the α-chain of C3 after residue 1290. This cleavage site is close but not identical to that of the human complement protease factor I, which attacks C3 activation fragments after residue 1298. The proximity of the cleavage sites of SplB and endogenous factor I suggested that SplB also degrades and inactivates endogenously generated C3 activation fragments. Indeed, Western blotting revealed that SplB cleaved C3b, iC3b, and C3c (Fig. S3). Again, SplB acted predominantly on the α-chains, while the β-chains of the three fragments remained intact.

### Serine protease inhibitors do not affect SplB-mediated C3 cleavage.

As SlpB has a serine protease motif, we attempted to inhibit the enzyme with the serine protease inhibitors phenylmethylsulfonyl fluoride (PMSF) and aprotinin. However, neither PMSF nor aprotinin (at 10 μM or 15 μM, respectively) affected SplB proteolysis (Fig. S4A and B). Moreover, SplB was remarkably heat stable, as some proteolytic activity for C3 was preserved even after incubation for 1 h at 65°C (Fig. S4).

### SplB cleaves C5 and terminal pathway proteins C6, C7, C8, and C9.

Complement actions at the C5 convertase level and via the terminal lytic pathway mediate essential effector functions. The cleavage of C5 generates the potent anaphylatoxin C5a and initiates the lytic terminal pathway via C5b. SplB also cleaved the α-chain of C5, generating fragments of around 40 to 60 kDa. The effect was concentration dependent (Fig. S4A). Determination of the N termini of the fragments via mass spectrometry revealed one dominant cleavage site after glutamine 1130 (Table 1 and Fig. 2D). However, SplB did not cleave the β-chain of C5. Thus, bacterial SplB also inhibits the complement cascade at the level of C5.

In addition, SplB cleaved and processed the four terminal complement components C6 (Fig. S5B), C7 (Fig. S5C), C8 (Fig. S5D), and C9 (Fig. S5E). The proteolysis of all terminal complement complex proteins was concentration dependent.

### SplB cleaves factor B and blocks the human alternative complement pathway.

To assess whether SplB interferes with the initiation of the alternative complement pathway, we tested the cleavage of the alternative pathway activator protease factor B and the complement inhibitor factor H. SplB cleaved factor B in a concentration-dependent manner (Fig. S6A). In contrast, factor H remained intact when incubated with SplB (Fig. S6B). Thus, SplB processes human C3 and factor B, two components of the alternative complement pathway, but not the central inhibitor factor H. This selectivity for effector proteins, but not for the regulator of the alternative pathway, shows that SplB contributes to immune evasion.

**FIG 3:**
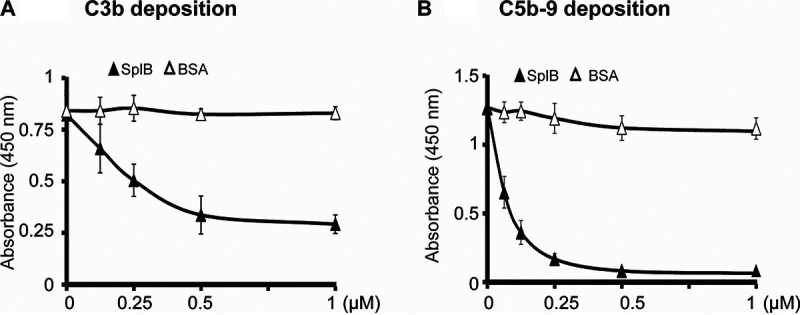
SplB inhibits the alternative complement pathway. To probe the spontaneous activation of the alternative pathway, microtiter plates were coated with LPS. Increasing concentrations of SplB or BSA were incubated with appropriately diluted NHS as the source of complement for 1 h, and the mixtures were added to the microtiter plates. After incubation for 20 min at 37°C, the plates were washed, and deposited C3b or C5b-9 was determined with specific antisera or monoclonal antibodies. SplB but not BSA diminished the deposition of C3b (A) and C5b-9 (B).

As SplB cleaves components of the activated C3 convertase of the alternative pathway but not the inhibitor factor H, we evaluated how SplB interferes with the function of the alternative pathway. Using an enzyme-linked immunosorbent assay (ELISA) with lipopolysaccharide (LPS)-coated microtiter wells, complement activation was monitored and quantified by C3b and C5b-9 deposition. SplB was incubated with complement-active NHS (20%), and the mixture was then added to LPS-coated microtiter wells to activate the alternative complement pathway. In this setting, SplB blocked C3b deposition. This effect was concentration dependent, and SplB at a concentration of 0.5 μM abolished C3b deposition (Fig. 3A). SplB also prevented C5b-9 deposition, again in a concentration-dependent manner. SplB at 0.5 μM inhibited C5b-9 deposition completely (Fig. 3B). Thus, SplB blocks the alternative pathway at the C3 and terminal complement complex C5b-9 (also termed the membrane attack complex) levels.

### SplB also cleaves components of the classical and lectin complement pathways.

**FIG 4:**
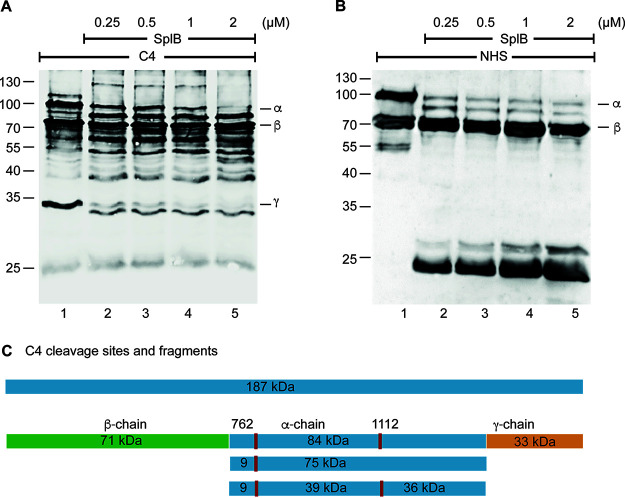
SplB-generated cleavage patterns of the complement protein C4. (A) Increasing concentrations of SplB were incubated with purified C4, the reaction mixtures were subjected to SDS-PAGE, and the cleavage patterns were visualized by Western blotting. SplB cleaved the α-chain of C4 in a concentration-dependent manner. (B) Increasing concentrations of SplB were incubated with complement-active NHS (0.5%), and the reaction mixture was separated by SDS-PAGE. Western blotting shows concentration-dependent cleavage of C4 by SplB. (C) Cleavage sites of C4, represented as described in the legend of Fig. 2C and D.

Next, we asked whether SplB can process components of the classical and lectin pathways. Indeed, SplB cleaved C4; both the α-chain and γ-chain, but not the β-chain, were processed into numerous fragments (Fig. 4A). Cleavage was concentration and time dependent (Fig. 4A; Fig. S7A). When incubated with the intact C4 present in NHS, SplB generated three major fragments, with mobilities of approximately 85, 30, and 23 kDa (Fig. 4B).

N-terminal peptides of C4 cleavage fragments were again identified by mass spectrometry. SplB hydrolyzed the α-chain of C4 after amino acids 762 and 1112 (Fig. 4C). The N-terminal sequences of the fragments showed that SplB cleaved after glutamine at position P1; at position P1′, glutamic acid and alanine were present, but amino acids at positions P2, P3, P4, and P5 were less conserved (Table 1). Similar to C3, SplB-mediated cleavage of C4 showed thermostability at 65°C (Fig. S7B). SplB also degraded C4b, the activation fragment of C4, as well as the classical and lectin pathway protease C2 (Fig. S7C and D).

### Human C1q and C4BP are not cleaved by SplB.

SplB processed neither C1q, the initiator of the classical pathway, nor C4BP, the inhibitor of the classical and lectin pathways. Both human proteins remained intact when incubated with SplB (Fig. S8A and B), again showing the specificity of the bacterial protease SplB.

### SplB blocks lectin and classical pathway activation.

**FIG 5:**
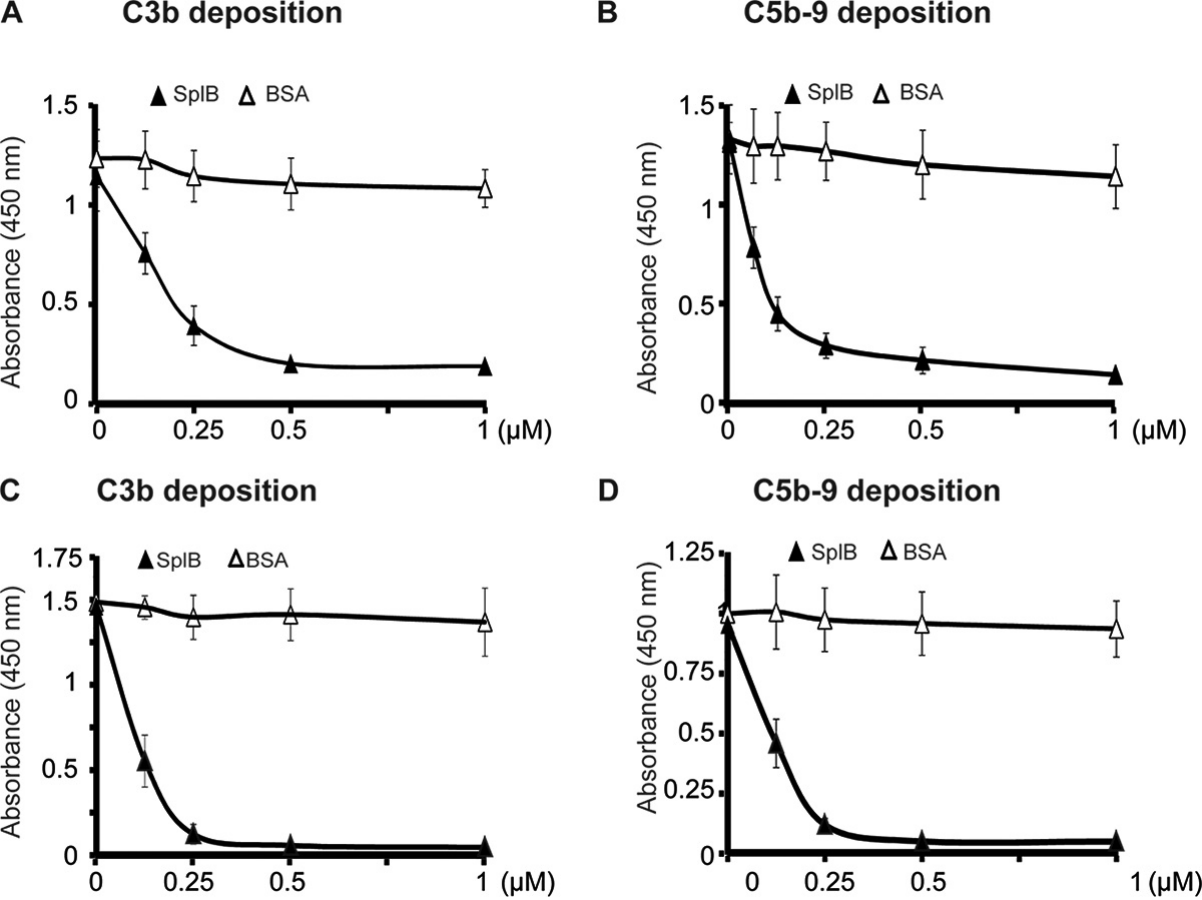
SplB inhibits the lectin and classical complement pathways. The effects of SplB on complement activation and the deposition of C3b and C5b-9 were analyzed as described in the legend to Fig. 3. (A and B) The lectin pathway was activated via mannan-coated microtiter plates. SplB interfered with the lectin pathway in a concentration-dependent manner, lowering the deposition of C3b (A) and C5b-9 (B). (C and D) To test for interference with the classical pathway, the microtiter wells were coated with IgM. Again, SplB reduced the classical pathway-mediated deposition of C3b (C) and C5b-9 (D) concentration dependently.

Next, we tested whether SplB blocks classical and/or lectin pathway activation. For this study, pathway-specific conditions were used by coating microtiter wells with relevant initiators. To generate activation conditions for the classical pathway, microtiter plates were coated with IgM. SplB was added to NHS (2%) as the complement source, and after 1 h at 37°C, the mixture was added to microtiter wells. After further incubation (20 min at 37°C), the levels of deposited C2, C4b, and C3b, as well as those of C5b-9, were measured. SplB did not inhibit C2 deposition (Fig. S9A); however, the enzyme interfered with C4b, C3b, and C5b-9 deposition. At 1 μM, SplB inhibited C4b deposition by 71% (Fig. S9B), and C3b and C5b-9 depositions were almost abolished at 0.5 μM SplB (Fig. 5A and B).

Similarly, the effect of SplB on the lectin pathway was studied using mannan-coated wells. Again, SplB did not inhibit C2 deposition (Fig. S9C) but blocked C4b deposition (Fig. S9D), C3b deposition, as well as C5b-9 deposition (Fig. 5C and D). In this lectin pathway setting, SplB at 0.5 μM blocked C4b deposition and almost abrogated C3b and C5b-9 deposition.

Thus, SplB blocked each of the three complement pathways. Evidently, SplB targeted several complement activation components and could act separately on C3, C4, and C5. Independently, SplB blocked the terminal pathway by cleaving each component of the lytic pathway, i.e., C6, C7, C8, and C9.

### SplB blocks complement deposition on the surface of S. aureus.

**FIG 6:**
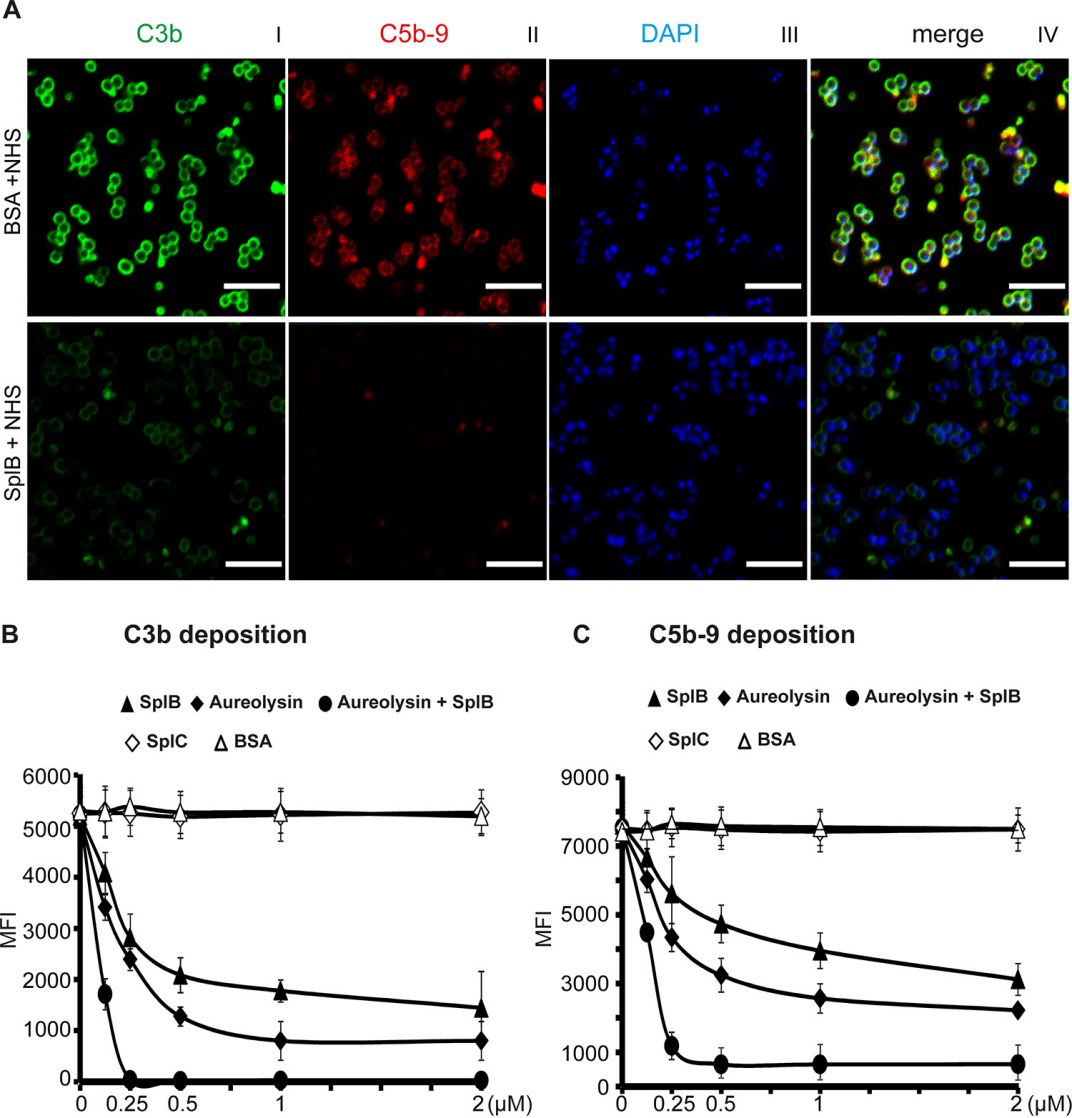
SplB and aureolysin reduce the deposition of C3b and C5b-9 on the surface of S. aureus cells. (A) SplB or BSA (1 μM) was incubated with complement-competent NHS (5%) for 1 h, and the mixture was added to *spa*-deficient S. aureus cells (RN1HF Δ*spa*) for 20 min at 37°C. C3b or C5b-9 on the bacterial cell surface was stained with specific fluorescent antibodies and evaluated by microscopy. In the control samples (BSA), there was abundant deposition of C3b (I, top, green) and C5b-9 (II, top, red) on the bacterial surfaces. SplB strongly interfered with complement deposition (C3b [I, bottom, green] and C5b-9 [II, bottom, red]). Panels III show bacterial DNA stained with 4′,6-diamidino-2-phenylindole (DAPI), and panels IV show the overlays of the three images. Bars, 100 μm. (B and C) SplB, aureolysin, or both were incubated with complement-active NHS (5%) for 1 h at the indicated concentrations. SplC and BSA served as controls. The resulting solutions were added to *spa*-deficient S. aureus cells (RN1HF Δ*spa*) for 20 min at 37°C. After washing, the deposition of C3b (B) or C5b-9 (C) was stained with specific antibodies and fluorescent secondary reagents and evaluated by flow cytometry. SplB and aureolysin inhibited the deposition of C3b and C5b-9 on the bacterial surface in a concentration-dependent manner. The combination of SplB and aureolysin had the strongest effect, completely abolishing the deposition of C3b (B) and reducing the deposition of C5b-9 by 90% (C). SplC and BSA, the negative controls, had no influence on complement deposition (B and C). Depicted are means ± SD from three independent experiments.

To study how pathogens might benefit from the multiple inhibitory actions of SplB, we first challenged S. aureus (strain RN1HG Δ*spa*) with complement-active NHS. This resulted in abundant C3b surface deposition (Fig. 6AI, top). C3b was evenly distributed on bacterial surfaces. In contrast, terminal pathway-mediated C5b-9 deposits showed a more clustered distribution, with large C5b-9 complexes at the bacterial cell poles or near the division septa (Fig. 6AII, top). SplB reduced C3b and also C5b-9 surface deposition (Fig. 6AI and II, bottom). When quantified by flow cytometry, SplB at 1 μM inhibited C3b deposition by 66% (SplB mean fluorescence intensity [MFI] = 1,777 ± 215; NHS MFI = 5,202 ± 515). This effect was specific; neither the staphylococcal protein SplC, which was also expressed in B. subtilis, nor bovine serum albumin (BSA) affected complement action (Fig. 6B). In addition, C5b-9 deposition on the surface of S. aureus cells was reduced by 48% (MFI of 3,950 ± 517, versus NHS MFI of 7,611 ± 660 [100%]) when 1 μM SplB was present in the culture. Again, neither SplC nor BSA blocked C5b-9 formation (Fig. 6C). Thus, SplB interferes with C3b and C5b-9 deposition on the bacterial cell wall.

### SplB blocks phagocytosis of S. aureus by human neutrophils.

**FIG 7:**
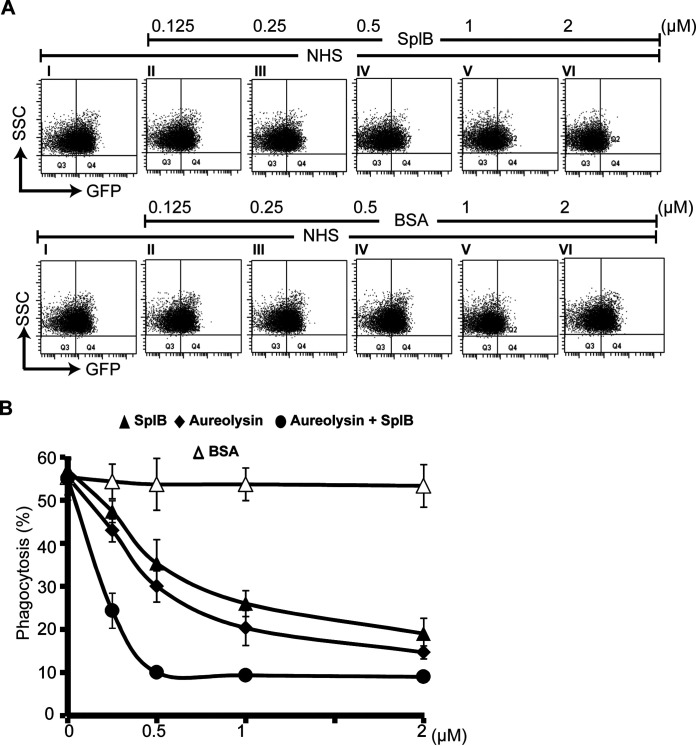
SplB and aureolysin interfere with the phagocytosis of S. aureus by neutrophils. SplB, aureolysin, both together, or BSA (control) at the indicated concentrations was incubated with complement-active NHS for 1 h. The mixture was added to GFP-expressing, *spa*-deficient S. aureus cells in the presence of human neutrophils for 20 min at 37°C. The phagocytosis of S. aureus by the neutrophils was analyzed by flow cytometry based on side scatter (SSC) and GFP positivity. (A) SplB inhibited phagocytosis in a concentration-dependent manner (top), while BSA had no influence (bottom). (B) Statistical analysis of the concentration-dependent inhibition of phagocytosis by the staphylococcal proteases. Both SplB and aureolysin alone interfered with the engulfment of the green fluorescent S. aureus cells by human neutrophils. Together, the bacterial proteases blocked opsonophagocytosis almost completely. The control protein BSA did not change the neutrophils’ phagocytic activity. Panel B represents means ± SD from three separate experiments.

Next, we asked whether SplB influences the complement-mediated opsonophagocytosis of bacteria. To this end, green fluorescent protein (GFP)-expressing S. aureus cells were challenged with complement-active NHS and incubated with human neutrophils. Using flow cytometry, neutrophils were identified by their granularity, and phagocytosed bacteria were identified by fluorescence (Fig. 7A). In this setting, 57% (57% ± 3%) of human neutrophils had phagocytosed bacteria after 20 min of coincubation. This fraction was reduced to only 19% (±4%) when SplB was added to human complement-active NHS (Fig. 7B). Thus, SplB blocks the complement-mediated opsonophagocytosis of S. aureus by human neutrophils.

### SplB cooperates with aureolysin.

In addition to SplB, S. aureus expresses another bacterial C3-cleaving protease, i.e., aureolysin. Aureolysin processes the α-chain of C3. This enzyme cleaves after residue 750N and generates a C3a-like (C3aL) and a C3b-like (C3bL) fragment. The latter is further processed by the human protease factor I (24).

Given that the first of the three SplB cleavage sites in C3 precisely corresponds to the cleavage motif of aureolysin (Fig. 2C and Table 1), we analyzed the contribution of the two bacterial proteases to complement and immune evasion and assessed their cooperation. To this end, C3 cleavage was evaluated using each protease alone or both proteases together. Both SplB and aureolysin cleaved C3. As expected, SplB generated more cleavage fragments than did aureolysin. Both proteases together had a more pronounced effect, and SplB further processed the C3bL fragment generated by aureolysin (Fig. S10A). Thus, the two bacterial proteases cooperated in C3 inactivation. C4, in contrast, was cleaved only by SplB and not by aureolysin, showing a broader substrate profile of SplB (Fig. S10B).

### SplB and aureolysin cooperate in complement and immune evasion.

We next asked whether the two bacterial proteases act in synergy when mediating complement and immune evasion. To this end, SplB and/or aureolysin was coincubated in complement-active NHS. Intact S. aureus cells were added, and following further incubation, the levels of deposited C3b were measured on the bacterial surface. SplB or aureolysin alone at 1 μM reduced C3b deposition by 66% or 85%, respectively. In combination, SplB and aureolysin (at 0.5 μM each) blocked C3b deposition completely (Fig. 6B).

Cooperativity was further evaluated for C5b-9 deposition. SplB or aureolysin alone (1 μM) reduced C5b-9 formation by 48% or 75% (SplB MFI = 3,950 ± 517; aureolysin MFI = 2,656 ± 427; NHS MFI = 7,611 ± 660 [100%]). Both enzymes together, each at 0.5 μM, reduced C5b-9 deposition by 90% (MFI = 717 ± 413) (Fig. 6C). This shows the cooperativity of the two bacterial proteases in blocking opsonization.

Finally, we asked how the two bacterial proteases affect the opsonophagocytosis of S. aureus by human neutrophils. Again, SplB and/or aureolysin was incubated with complement-active NHS for 1 h. Next, human neutrophils were added together with GFP-expressing S. aureus. After a 20-min incubation, phagocytosis was quantified by flow cytometry (Fig. 7A). In the absence of bacterial enzymes, 57% ± 3% of the neutrophils incorporated S. aureus. SplB (2 μM) decreased phagocytosis to 19% ± 4% (Fig. 7B), and SplB together with aureolysin (0.5 μM each) reduced phagocytosis to background levels (9%). Thus, SplB can efficiently block complement-mediated opsonophagocytosis of S. aureus by human neutrophils.

## DISCUSSION

Clinical S. aureus isolates secrete serine protease-like proteins, termed Spl proteins. The number of *spl* genes varies, and up to six of them are organized in a single operon encoding the proteins SplA to SplF. Little is known about the functions of the individual proteins and their roles in pathogen-host interactions. Here, we demonstrate that SplB, one member of this cluster, is a protease that cleaves and inactivates several host complement proteins. By degrading the central human complement components C3, C3b, C4, and C4b as well as factor B, SplB can inhibit each of the three complement pathways. Furthermore, SplB can cleave the components of the terminal complement pathway, C5, C6, C7, C8, and C9, thereby interfering with complement at multiple levels. Significantly, SplB was active in human serum and effectively reduced the deposition of C3b and C5b-9 on the surface of S. aureus. Consequentially, SplB strongly diminished the uptake and phagocytosis of bacteria by human neutrophils. Remarkably, SplB did not detach or degrade the soluble complement regulator factor H or C4BP. Thus, SplB selectively targets complement activation and functions as a bacterial complement evasion factor.

Proteolytic cleavage and inactivation of complement proteins are common mechanisms of immune escape that are used by many human pathogens (37–41). Many pathogenic microbes express and secrete proteases that target C3, the central complement protein. These proteases include Pra1 from Candida albicans, Alp1 and Mep1 from Aspergillus fumigatus, Nalp1 from Neisseria meningitidis, the group A streptococcal cysteine protease SpeB (42), aureolysin from S. aureus (24), and GelE from Enterococcus faecalis (45). Escherichia coli secretes proteases with other profiles: Pic inactivates C2, C3, and C4, while EspP degrades C3, C3b, and C5 (43).

pH-regulated antigen 1 (Pra1) from C. albicans cleaves the α-chain of C3 6 residues away from the endogenous C3 convertase cleavage site, generating inactive C3aL and C3bL molecules, thereby blunting host complement attack (40). Similarly, NalP of N. meningitidis and the thermolysin of *Leptospira* strains cleave the α-chain of C3 4 residues away from the host C3 convertase cleavage site (39, 44). In contrast, aureolysin, the only other known complement-targeting protease of S. aureus, as well as GelE of E. faecalis cleave the α-chain of C3 2 residues downstream of the host C3 convertase site, generating active C3aL and C3bL fragments (24, 45). Fragments generated by pathogen-encoded proteases can be further degraded by host regulators, i.e., factor H and factor I, for inactivation.

SplB cleaved the α-chains of C3, C4, and C5 and generated smaller fragments than did aureolysin. Thus, SplB can abolish complement effector functions directly, independent of host-derived complement regulators. Moreover, SplB further degraded the C3bL product generated by aureolysin. In effect, SplB and aureolysin can act in combination or even in synergy with each other and with human complement inhibitors.

Several infectious agents also attack terminal complement pathway proteins; however, in this case, mechanisms of immune evasion are less well understood than those for C3 degradation. Although Gram-positive bacteria are protected from C5b-9-mediated lysis by a thick peptidoglycan layer, Gram-positive bacteria also secrete proteins that inhibit C5b-9 formation (46). The terminal complex targets membranes and forms pores, and in addition, the soluble C5b-9 complex can bind to endothelial cells and induce proinflammatory and procoagulant activities (47). The streptococcal inhibitor of complement (SIC) from Streptococcus pyogenes inhibits C5b-9 formation, enhancing bacterial survival in human serum and during murine infection (48). The cysteine protease of Porphyromonas gingivalis (49) and EspP of E. coli directly cleave and inactivate C5 (43).

SplB of S. aureus also cleaves and inactivates C5, generating several smaller fragments. Moreover, SplB cleaves C6, C7, C8, and C9 proteins, and this inactivation decreased C5b-9 deposition on the surface of S. aureus. SplB is active in human serum, and the bacterial protein effectively blocked C3b and C5b-9 deposition on the surface of S. aureus cells. SplB acted in cooperation or even in synergy with aureolysin, and at concentrations of 500 nM each, the proteases completely inhibited the deposition of C3b as well as that of C5b-9 on the bacterial surface. This concerted action efficiently blocked opsonophagocytosis of the bacteria by neutrophils.

Although SplB attacks the complement cascade at numerous stages and sites, SplB-mediated proteolysis is clearly selective, which distinguishes this bacterial virulence determinant from protein-degrading enzymes with a primary nutritional function. For example, the α-chains in complement proteins C3 and C4 were selectively cleaved, while the β-chains remained intact. And, intriguingly, SplB spared the human complement inhibitors factor H and C4BP. Thus, SplB’s multipronged attack on the human complement system selectively targets complement activation. Further evidence for the function of SplB, and likely also that of other Spl proteins, as a virulence determinant is the location of the *spl* operon in the pathogenicity island νSaβ (29, 50). The transcription of this operon is strongly upregulated by the global virulence regulators SaeR, MgrA, and SarA (51). Another indication is the proteases’ narrow substrate ranges, which appear to be unique for each Spl (31, 32, 34, 36, 52).

Mapping of SplB cleavage sites in complement proteins featured glutamine at position P1 in five of six motifs, and in three sites, there was a leucine at position P2. This is in good agreement with previous studies using peptide libraries and synthetic peptides, which defined Trp-Glu-Leu-Gln (P4 to P1) as the preferred cleavage motif of SplB (34, 35).

SplB contains the canonical catalytic triad that defines serine proteases of the chymotrypsin family (34); however, SplB enzymatic activity was not affected by the serine-specific protease inhibitors aprotinin and PMSF. These results confirmed the findings of Dubin et al. that SplB protease activity was not inhibited by serine protease inhibitors when synthetic peptides were used as the substrates (53). Several serine protease inhibitors are present in human plasma or serum (54), but SplB was active in NHS and cleaved C3 and C4. Therefore, we speculate that S. aureus has evolved a molecular mechanism to resist host serine protease inhibitors so that the bacteria can successfully inactivate serum proteins and survive in the host.

Not all S. aureus strains can produce SplB. We found that the composition of the *spl* operon was closely associated with the genetic background of S. aureus strains. In our study, using bacteria isolated from healthy Belgian volunteers, the *splB* gene was present in around half of the human nasal S. aureus isolates, comprising most of the dominant clonal lineages. However, two major clonal lineages, CC30 and CC45, lack *splB*. Overall, the complete *spl* operon was present in 18.8% of S. aureus nasal isolates, while only 17.7% lacked any of the *spl* genes, among them the CC45 strains. These findings are in good agreement with a previous report about a diverse collection of S. aureus clinical isolates, underscoring the high prevalence of this group of virulence factors (52).

In conclusion, SplB is an S. aureus virulence determinant that cleaves many human complement proteins and inhibits all three complement activation pathways, functioning as a multipronged immune evasion factor. This enzyme is thus part of the concerted action by which S. aureus counteracts the complement system and neutrophils (reviewed in references 8 and 10). The crucial importance of opsonization and phagocytosis in the defense against S. aureus is highlighted by the pronounced redundancy of numerous immune evasion mechanisms targeting these immune effector mechanisms.

## MATERIALS AND METHODS

### S. aureus isolates.

S. aureus was isolated from nasal swabs of 96 healthy volunteers in the Department of Otorhinolaryngology, Ghent University, in 2011 and stored as glycerol stocks. The volunteers’ age was 25.5 ± 4.9 years (mean ± standard deviation [SD]); 60 were female, and 36 were male. All individuals gave informed consent (approved by the ethical board of the University of Ghent [EC2011/612-B670201112093]). Following incubation overnight on blood agar, a single colony was selected, confirmed to be S. aureus using the Slidex Staph Plus coagulase test kit (bioMérieux), grown in 3 mL lysogeny broth (LB) medium for 5 h, and pelleted. DNA was isolated, and the S. aureus strains were molecularly *spa* typed as described previously (55). Briefly, the variable region of the *spa* gene was amplified using the primers shown in Table S1 in the supplemental material, sequenced, and grouped into clonal clusters (CCs) and sequence types (STs) using Ridom StaphType software (Ridom GmbH, Würzburg, Germany). The *spl* operon was analyzed using two multiplex PCR systems with the primers shown in Table S1 and Dream *Taq* DNA polymerase (Thermo Fisher Scientific, Waltham, MA, USA) under the conditions shown in Table S2. DNA of the S. aureus strain USA300 whose *spl* operon contains all genes *splA* to *splF* served as the positive control, and DNA of E. coli BL21 served as the negative control.

### Serum, proteins, and antibodies.

Normal human serum (NHS) was collected from healthy individuals upon informed consent, pooled, and stored at −80°C until use. Human plasma-purified complement proteins C1q, C2, C3, C3b, C4, C4b, C5, C5b, C6, C7, C8, and C9; factor H; and C4BP were purchased from CompTech (Complement Technology Inc.) (Tyler, TX, USA). Goat antisera against human C1q, C2, C3, C4, C5, C6, C7, C8, C9, and factor H were procured from CompTech (Tyler, TX, USA). Sheep polyclonal anti-human C4BP antiserum was purchased from Abcam. Horseradish peroxidase (HRP)-conjugated rabbit anti-goat immunoglobulins and HRP-conjugated goat anti-rabbit immunoglobulins were purchased from Dako Deutschland GmbH (Hamburg, Germany).

### Expression and purification of Spl proteins.

Spl proteins were recombinantly expressed and purified as described previously (27). In brief, B. subtilis 6051HGW LS8P-D, which is deficient for the proteases AprE, Bpr, Epr, Mpr, NprB, NprE, Vpr, and WprA, was used as the expression host. Culture supernatants containing native Spls were subjected to tangential-flow filtration, and buffer was exchanged with 20 mM Tris-HCl (pH 7.5). The Spl proteins were then purified by ion-exchange chromatography with an SP Sepharose column (GE Healthcare), followed by two-step size exclusion centrifugation with centrifugal filter units (Amicon Ultra 30K/10K; Merck). Thus, the Spl solution was sterile filtered, and the buffer was exchanged with phosphate-buffered saline (PBS). The quality of the purified proteins was verified by SDS-PAGE, and the protein concentration was determined by spectrophotometry.

### Cleavage assays.

SplB at the indicated concentrations was added to purified human complement proteins (C1q, C2, C3, C3b, C4, C4b, C5, C5b, C6, C8, C9, factor H, and C4BP [1 μg of each]) or NHS (0.5%) in 20 mM HEPES buffer (pH 7.4) (50-μL total volume), and the mixtures were incubated for 2 h at 37°C. The reaction was stopped by the addition of 20 μL of Roti Load 1, and the samples were boiled (10 min at 95°C) and separated by SDS-PAGE. Next, the proteins were transferred to a membrane, and their cleavage products were visualized by Western blotting using specific antisera. To attempt to inhibit the protease activity of SplB, the serine protease inhibitors phenylmethylsulfonyl fluoride (PMSF; Roth, Germany) and aprotinin (Sigma, Germany) were used. Briefly, PMSF (at concentrations of 2.5, 5, and 10 μM) or aprotinin (3.75, 7.5, and 15 μM) was added to SplB with C3 or C4 (1 μg each), and the mixtures were incubated for 2 h at 37°C. The cleavage patterns were assessed by Western blotting as described above.

### N-terminal peptide sequences of the SplB-generated cleavage fragments of C3, C4, and C5.

The identification of peptide N termini after the cleavage of C3, C4, and C5 by SplB was done using the terminal amine isotopic labeling of substrates (TAILS) method described previously by Kleifeld and colleagues (56) according to version 4 of the protocol available at https://clip.ubc.ca/resources/protocols-and-sops/.

In brief, 10 μg of either C3, C4, or C5 was incubated at 37°C with 10 μg of SplB alone or SplB and aureolysin, generating cleaved samples. Samples without the addition of enzymes served as controls. The reaction was stopped by freezing the samples. The samples were lyophilized and reconstituted in 4 M guanidinium chloride in 100 mM HEPES (pH 7.5) before reduction and alkylated by the addition of dithiothreitol (DTT) and iodoacetamide (IAA) to final concentrations of 5 mM and 15 mM, respectively. The samples were dimethylated using ^12^CH_2_O (Sigma, Germany) for the cleaved samples and ^12^CD_2_O (Sigma, Germany) for the controls. After tryptic digestion of the cleaved samples or controls, the newly generated internal tryptic N termini were removed using hyperbranched polyglycerol-aldehydes (version 2) polymer. Thereafter, the peptide mixtures were desalted using ZipTip-μC_18_ tips (Millipore), concentrated by evaporation under a vacuum, and subsequently resolved in 0.1% acetic acid–2% acetonitrile (ACN) to be measured by mass spectrometry.

### Mass spectrometry.

Chromatographic separation of the peptides was performed on a reverse-phase nano-Acquity ultraperformance liquid chromatography (UPLC) column (1.7 μm, 100-μm internal diameter by 100 mm; Waters GmbH, Eschborn, Germany) using a 45-min nonlinear gradient ranging from 5 to 45% ACN in a 0.1% aqueous acetic acid solution at a flow rate of 400 nL/min. The nano-LC column was linked by electrospray ionization to an LTQ-Orbitrap Velos mass spectrometer (Thermo Scientific, San Jose, IL, USA). Precursor ions of an *m/z* range of 325 to 1,525 (*r* = 30.000) were subjected to data-dependent tandem mass spectrometry (MS/MS) fragmentation of the 20 most intense precursor peaks in the ion trap at a collision-induced energy (CID) of 35%. Repetitive MS/MS acquisition was eliminated by setting dynamic exclusion of 60 s for previously selected precursors (Tables S3 and S4).

Proteome discoverer software (version 2.2; Thermo Scientific, San Jose, IL, USA) was used to analyze the raw data. Ratios of the obtained peptide intensities were calculated, and peptide ratios between the cleaved sample and the control sample of >10 were considered significant. In the case of replicate experiments, the peptides had to be identified in all replicates. For details, see Tables S3 and S4 in the supplemental material.

### Complement activation.

To evaluate SplB effects on complement, SplB was added to complement-active NHS, and the mixture was then added to microtiter wells, which were coated to initiate one of the three complement pathways. Microtiter plates were coated with IgM (20 μg/mL; 100 μL/well), mannan (20 μg/mL), or LPS (20 μg/mL) in PBS overnight at 4°C. After washing, the plates were blocked with 3% BSA for 1 h at room temperature (RT).

To assess the inhibitory effect of SplB on the classical pathway or lectin pathway, SplB at increasing concentrations was added to NHS (100 μL of 2% NHS in 20 mM HEPES buffer [pH 7.4] containing 144 mM NaCl, 5 mM CaCl_2_, and 2.5 mM MgCl_2_) for 1 h at 37°C. BSA served as a negative control. After incubation, the SplB-NHS mixture was added to the coated microtiter plates, and the combination was incubated at 37°C for 20 min. Following washing, deposited C2 was evaluated with goat human C2 antiserum. Deposited C3b, C4b, or C5b-9 was detected with mouse monoclonal antibodies specific for C3b, C4b, or C5b-9, followed by horseradish peroxidase-conjugated goat anti-mouse IgG.

To evaluate the inhibitory effect of SplB on the alternative pathway, SplB at increasing concentrations (0.125, 0.25, 0.5, 1, and 2 μM) was incubated with NHS (100 μL of 20% NHS in 20 mM HEPES buffer [pH 7.4] containing 144 mM NaCl, 7 mM MgCl_2_ [20%], and 5 mM EGTA) at 37°C for 1 h. BSA served as a negative control. Next, the mixture was added to LPS-coated microtiter plates. Following incubation for 20 min at 37°C, deposited C3b and C5b-9 were detected with monoclonal antibodies specific for C3b and C5b-9.

### C3b and C5b-9 deposition on the surface of S. aureus.

The deposition of C3b and C5b-9 on the bacterial surface was studied by indirect immunofluorescence microscopy and flow cytometry. *spa*-deficient S. aureus cells (strain RN1HG Δ*spa*) were cultivated in Tris-buffered saline (TBS) medium overnight. The bacteria (200 μL) were then transferred to fresh TBS medium (10 mL) and further cultivated until the optical density at 600 nm (OD_600_) reached 1.0. After washing three times with Dulbecco's phosphate-buffered saline (DPBS), the bacteria (100 μL) were challenged with complement-active NHS that had been preincubated with SplB (or BSA) for 60 min at 37°C.

For microscopy, SplB, or BSA as a negative control, was added to 100 μL of NHS (5% NHS in 20 mM HEPES [pH 7.4]) at a final concentration of 1 μM. Following incubation for 1 h at 37°C, the mixture was diluted to 2.5% NHS with 100 μL HEPES (20 mM [pH 7.4] supplemented with 288 mM NaCl, 10 mM CaCl_2_, and 5 mM MgCl_2_). *spa*-deficient S. aureus cells (10^8^) were added, incubated for a further 20 min at 37°C, and washed. The bacteria were allowed to adhere to poly‐l‐lysine-coated coverslips for 30 min at RT, nonadherent cells were washed off, and the attached bacteria were fixed with 4% formaldehyde for 10 min. The coverslips were blocked with 3% BSA for 1 h at RT, washed, and incubated with goat anti-human C3 antiserum or monoclonal mouse anti-human C5b-9 antibodies. Antibody binding was visualized using Alexa Fluor 488-conjugated rabbit anti-goat IgG or Alexa Fluor 647-conjugated donkey anti-mouse IgG. The coverslips were mounted onto glass slides with Mount Fluor (Pro Taqs), and images were acquired with a laser scanning microscope (LSM 710; Zeiss, Germany) using ZEN 2009 software.

For flow cytometry, the same protocol was applied as the one for microscopy; however, the initial concentration of NHS was 10% (later diluted to 5%). After incubation with complement-active NHS that had been pretreated with SplB or BSA, the bacteria were stained in suspension, and their fluorescence intensity was measured by flow cytometry (LSR II; Becton, Dickinson). The median fluorescence intensity was calculated with FlowJo software (Becton, Dickinson).

### Isolation of human neutrophils.

Human neutrophils were isolated from buffy coats as previously described (57). In brief, dextran T 500 (3%) (Carl Roth GmbH, Germany) (3% dextran T 500 in 0.9% NaCl, at 4:1) was added to the buffy coats in 15-mL tubes, and erythrocytes were sedimented at gravity for 20 min. The white blood cell layer was removed carefully, and cells were pelleted by centrifugation at 200 × *g* for 20 min. The cell pellet was resuspended in 12 mL of Hanks’ balanced salt solution (HBSS; Lonza Cologne GmbH, Germany), layered on top of 3 mL Ficoll-plaque Plus (GE Healthcare, Germany), and centrifuged at 400 × *g* for 20 min. Next, residual erythrocytes were lysed in lysis buffer (150 mM NH_4_Cl, 10 mM KHCO_3_, and 10 mM EDTA [pH 7.4]). The neutrophils were washed (200 × *g* for 10 min), suspended in HBSS, and used immediately.

### Phagocytosis assay.

To examine the effect of SplB on phagocytosis, the uptake of fluorescent bacteria by human neutrophils was monitored by flow cytometry as described previously. In brief, NHS served as a source of complement. GFP-expressing S. aureus RN1HG Δ*spa* was cultivated overnight in TBS containing tetracycline (20 μg/mL) and erythromycin (10 μg/mL). The bacteria were washed and resuspended in HBSS. SplB or BSA was incubated with NHS (100 μL of 10% NHS in 20 mM HEPES [pH 7.4]) for 1 h at 37°C and then diluted to 5% NHS by the addition of 100 μL of 20 mM HEPES (pH 7.4) containing 288 mM NaCl, 10 mM CaCl_2_, and 5 mM MgCl_2_. This mixture was added to freshly isolated neutrophils together with GFP-expressing S. aureus (multiplicity of infection [MOI] of 1:5), and phagocytosis was initiated at 37°C for 20 min. The reaction was stopped by adding ice-cold paraformaldehyde (100 μL; 4% solution), and phagocytosis was evaluated by determining granularity and GFP fluorescence of neutrophils using flow cytometry (BD LSR II). The percentage of granular neutrophils, which were fluorescence positive due to associated GFP-expressing bacteria, was used as a readout for phagocytosis.
