# Supplementary material for: The Protease SplB of Staphylococcus aureus Targets Host Complement Components and Inhibits Complement-Mediated Bacterial Opsonophagocytosis
Source: J Bacteriol. 2022 Jan 18;204(1):e00184-21. doi: 10.1128/JB.00184-21 (PMC8765433; doi:10.1128/JB.00184-21)
Supplement: Supplemental file 1 — Tables S1 to S3 and Fig. S1 to S10. Download JB.00184-21-s0001.pdf, PDF file, 7.2 MB [file jb.00184-21-s0001.pdf]

Supplementary Material

The Protease SplB of *Staphylococcus aureus* Targets Host Complement Components and Inhibits Complement-Mediated Bacterial Opsonophagocytosis

Prasad Dasari,[a] Maria Nordengrün,[b] Cláudia Vilhena,[a] Leif Steil,[c] Goran Abdurrahman,[b] Kristin Surmann,[c] Vishnu Dhople,[c] Julia Lahrberg,[b] Claus Bachert,[d*] Christine Skerka,[a] Uwe Völker,[c] Barbara M. Bröker,[b] Peter F. Zipfel[a,e]

[a]Department of Infection Biology, Leibniz Institute for Natural Product Research and Infection Biology, Hans Knöll Institute, Jena, Germany

[b]Institute of Immunology, University Medicine Greifswald, Greifswald, Germany

[3]Interfaculty Institute of Genetics and Functional Genomics, University Medicine Greifswald, Greifswald, Germany

[4]Upper Airways Research Laboratory, Department of Otorhinolaryngology, Ghent University, Ghent, Belgium; Claus.Bachert@UGent.be

[5]Institute of Microbiology, Friedrich Schiller University, Jena, Germany

Barbara M. Bröker and Peter F. Zipfel contributed equally as senior authors

Address correspondence to

Barbara M. Bröker, Department of Immunology, Institute of Immunology and Transfusion Medicine, University Medicine Greifswald, Greifswald, Germany, broeker@uni-greifswald.de,and

Peter F. Zipfel, Department of Infection Biology, Leibniz Institute for Natural Product Research and Infection Biology, Hans Knöll Institute, Beutenbergstr. 11a, 07745 Jena, Germany. Phone: +49 (0) 3641 532-1300; Fax: +49 (0) 3641 532-0807; E-mail: peter.zipfel@leibniz-hki.de

Abbreviations: ACN: acetonitrile; BSA: bovine serum albumin; C: complement protein; DTT: dithiothreitol IAA: iodoacetamide; NHS: normal human serum; Spl: Serine protease-like protein; TAILS: terminal amine isotopic labelling of substrates;

Running Title: *S. aureus* Protease SplB Targets Host Complement

Supplemental Tables

Table S1. Primers for the molecular characterization of the *S. aureus* nasal isolates

***Spa* typing**	*spa*	spa-5’	taa aga cga tcc ttc ggt gag c
		spa-3'	cag cag tag tgc cgt ttg ctt
***spl*-Multiplex 1**	*splA*	splA-1	taa tga tat ttt taa aaa tag agt
		splA-2	caa acc ttc tgt act tgt t
	splC	splC-1	atg aaa atg tcc aag cat t
		splC-2	ggc acc att ata ttc aga acc
	splD	splD-1	gaa att aat tac caa cac ga
		splD-2	tgc cag gtt gga caa cc
***spl*-Multiplex 2**	*splB*	splB-1	tga aga gcg tgc aat aga a
		splB-2	cag tat gcg ctg aat ata ca
	splE	splE-1	cgt tgc agg tat gga aat t
		splE-2	gcg gtt tcc acc aaa gtg
	splF	splF-1	taa aca aat tac aaa tac aaa
		splF-2	gat tac acc aat agc ttc gtg t

Table S2. Conditions for the *spl*-multiplex PCRs

**PCR Master mix**		
	**Multiplex 1**<br>***spl A, splC, splD***	**Multiplex 2**<br>***spl B, spl E, splF***
PCR H_2O	11,8 µl	11,8 µl
10x DreamTaq™ Green Buffer	2,5 µl	2,5 µl
dNTPs (1 mM)	2,5 µl	2,5 µl
$MgCl_2$ (25 mM)	2 µl	2 µl
Primers (5 µM)	0,75 µl (*splC1, splC2*, *splD1, splD2*), 1 µl (*splA1, splA2*)	0,75 µl (*splB1, splB2*, *splE1, splE2*, *splF1, splF2*)
DreamTaq™ DNA Polymerase	0,2 µl	0,2 µl
	**24 µl master mix + 1 µl DNA**	
**Thermocycling conditions**		
Denaturing	94 °C	5 min
30 cycles of		
Denaturing	94 °C	45 s
Annealing	60 °C	45 s
Elongation	72 °C	90 s
Final elongation	72 °C	7 min

Table S3: Nano LC-MS/MS data acquisition parameters.

**LC-Parameters**	
Instrument	NanoAcquity UPLC (Waters GmbH, Eschborn, Germany)
Trap column	NanoAcquity UPLC 2G-V/M trap Symmetry C18 pre-column, 2 cm length, 180 µm ID and 5 µm particle size (Waters GmbH, Eschborn, Germany)
Analytical column	NanoAcquity BEH130 C18 column, 10 cm length, 100 µM ID and 1.7 µm particle size (Waters GmbH, Eschborn, Germany)
Buffer system	Binary buffer system consisting of buffer A (0.5% DMSO in water with 0.1% acetic acid) and buffer B (5% DMSO in acetonitrile with 0.1% acetic acid)
Flow rate	400 nl/min
Gradient	0 min 1% B, 2 min 5% B, 30 min 45% B, 32 min 90% B, 34 min 1% B, 40 min 1% B
Column oven temperature	40°C
**MS-Parameters**	
Instrument	LTQ-Orbitrap Velos mass spectrometer (Thermo Electron Corporation, Germany)
Ion Source	Nano-ESI source and installed with a Picotip Emmitter (New Objective, USA).
Operation mode	Data-dependent acquisition
**Full MS-Parameters**	
MS scan resolution	30,000
AGC target	1e6
Max. ion injection time for the MS scan	10ms
Scan range	325 to 1525 m/z
Spectra data type	Profile
**MS2-Parameters**	
MS/MS AGC target	1e4
Max. ion injection time for the MS/MS scans	100 ms
Selection for MS/MS	20 most abundant isotope patterns with charge ≥ 2 from the survey scan
Isolation width	2 m/z
Dissociation mode	collision-induced dissociation (CID)
Normalized collision energy	35%
Dynamic exclusion	60 s
Spectra data type	centroid
Charge exclusion	Unassigned, 1, 4, and above

Table S4: SplB Generates C3 Cleavage Fragments.

Protein Acce	Annotated Sequence	Modifications in Master Proteins	Positions in Master	Start	# Protein Groups	# Proteins	# PSMs	# Missed Cleavages	XCorr Sequest HT	Ions Score Mascot	Ratio (C3-SplB / C3)	Area C3-SplB	Area C3*
P01024	[R].LESEETMVLEAH.[D]		P01024 [36-47]	36	1	1	2	0	3.57	29.64	**43.75**	3.50E+06	8.00E+04
P01024	[Q].SGYLFIQTDKTIYTPGSTVLYR.[I]		P01024 [127-148]	127	1	1	2	0	5.33	47.41	**20.00**	1.60E+06	8.00E+04
P01024	[Q].SGYLFIQTDKTIYTPGSTVLYR.[I]	P01024 2×Dimethyl [K136;]	P01024 [127-148]	127	1	1	2	0	5.31	51.95	**20.00**	1.60E+06	8.00E+04
P01024	[Q].TDKTIYTPGSTVLYR.[I]		P01024 [134-148]	134	1	1	2	0	3.58	27.22	**12.77**	8.30E+07	6.50E+06
P01024	[Q].TDKTIYTPGSTVLYR.[I]	P01024 2×Dimethyl [K136;]	P01024 [134-148]	134	1	1	2	0	3.52	41.07	**12.77**	8.30E+07	6.50E+06
P01024	[Q].DSLSSQNQLGVLPLSWDIPELVNMGQWKIR.[A]	P01024 2×Dimethyl [K205;]	P01024 [178-207]	178	1	1	4	1	4.17	67.41	**33.75**	2.70E+06	8.00E+04
P01024	[R].IPIEDGSGEVVLSR.[K]		P01024 [291-304]	291	1	1	2	0	2.45	29.52	**14.05**	5.90E+07	4.20E+06
P01024	[D].GSGEVVLSR.[K]		P01024 [296-304]	296	1	1	2	0	2.77	48.78	**36.36**	3.20E+06	8.80E+04
P01024	[Y].VSATVILHSGSDMVQAER.[S]		P01024 [326-343]	326	1	1	2	0	4.31	37.34	**15.00**	1.20E+06	8.00E+04
P01024	[Q].GEDTVQSLTQGDGVAK.[L]	P01024 2×Dimethyl [K408;]	P01024 [393-408]	393	1	1	1	0		89.98	**50.00**	4.00E+06	8.00E+04
P01024	[Q].ELSEAEQATR.[T]		P01024 [430-439]	430	1	1	2	0	3.21	37.63	**45.00**	3.60E+06	8.00E+04
P01024	[Q].ALPYSTVGNSNNYLHLSVLR.[T]		P01024 [443-462]	443	1	1	4	0	5.45	94.04	**14.29**	2.00E+07	1.40E+06
P01024	[R].LVAYYTLIGASGQR.[E]		P01024 [531-544]	531	1	1	4	0	4.12	60.49	**12.79**	7.80E+06	6.10E+05
P01024	[Y].YTLIGASGQR.[E]		P01024 [535-544]	535	1	1	2	0	2.98	45.67	**55.00**	4.40E+06	8.00E+04
P01024	[F].LDCCNYITELR.[R]		P01024 [725-735]	725	1	1	2	0	3.28	53.76	**31.25**	2.50E+06	8.00E+04
P01024	[D].CCNYITELR.[R]		P01024 [727-735]	727	1	1	1	0	2.49	28.48	**45.16**	1.40E+08	3.10E+06
P01024	[R].ASHLGLAR.[S]		P01024 [741-748]	741	1	1	1	0	2.54	36.37	**10.75**	8.60E+05	8.00E+04
P01024	[R].SNLDEDIIAEENIVSR.[S]		P01024 [749-764]	749	1	1	34	0	6.01	125.43	**52.50**	4.20E+06	8.00E+04
P01024	[N].LDEDIIAEENIVSR.[S]		P01024 [751-764]	**751**	1	1	8	0	4.74	80.46	**39.39**	2.60E+07	6.60E+05
P01024	[D].EDIIAEENIVSR.[S]		P01024 [753-764]	**753**	1	1	39	0	3.88	80.06	**2000.00**	1.60E+08	8.00E+04
P01024	[D].IPPADLSDQVPDTESETR.[I]		P01024 [962-979]	962	1	1	4	0	3.52	66.56	**10.13**	8.10E+05	8.00E+04
P01024	[Q].GTPVAQMTEDAVDAER.[L]		P01024 [984-999]	**984**	1	1	14	0	5.49	109.57	**837.50**	6.70E+07	8.00E+04
P01024	[Q].GTPVAQMTEDAVDAER.[L]		P01024 [984-999]	**984**	1	1	4	0	4.84	107.30	**26.25**	2.10E+06	8.00E+04
P01024	[Q].MTEDAVDAER.[L]		P01024 [990-999]	990	1	1	7	0	3.53	64.31	**1625.00**	1.30E+08	8.00E+04
P01024	[Q].MTEDAVDAER.[L]		P01024 [990-999]	990	1	1	1	0	2.09	37.90	**31.25**	2.50E+06	8.00E+04
P01024	[Q].EDAPVIHQEMIGGLR.[N]		P01024 [1120-1134]	1120	1	1	9	0	4.13	67.99	**250.00**	2.00E+07	8.00E+04
P01024	[Q].EDAPVIHQEMIGGLR.[N]		P01024 [1120-1134]	1120	1	1	2	0	3.55	44.65	**20.00**	1.60E+06	8.00E+04
P01024	[E].DAPVIHQEMIGGLR.[N]		P01024 [1121-1134]	1121	1	1	2	0	3.23	47.41	**16.25**	1.30E+06	8.00E+04
P01024	[D].APDHQELNLDVSLQLPSR.[S]		P01024 [1286-1303]	1286	1	1	2	0	3.65	32.12	**10.00**	8.00E+05	8.00E+04
P01024	[Q].ELNLDVSLQLPSR.[S]		P01024 [1291-1303]	**1291**	1	1	6	0	4.98	79.65	**122.50**	9.80E+06	8.00E+04
P01024	[N].LDVSLQLPSR.[S]		P01024 [1294-1303]	**1294**	1	1	7	0	3.20	56.59	**2500.00**	2.00E+08	8.00E+04
P01024	[D].VSLQLPSR.[S]		P01024 [1296-1303]	**1296**	1	1	6	0	2.78	57.03	**3875.00**	3.10E+08	8.00E+04
P01024	[R].IHWESASLLR.[S]		P01024 [1311-1320]	1311	1	1	2	0	3.19	37.56	**27.50**	2.20E+06	8.00E+04
P01024	[Q].DAKNTMILEICTR.[Y]	P01024 2×Dimethyl [K1381;]	P01024 [1379-1391]	1379	1	1	13	0	4.29	50.71	**825.00**	6.60E+07	8.00E+04
P01024	[S].DDKVTLEER.[L]	P01024 2×Dimethyl [K1526;]	P01024 [1524-1532]	1524	1	1	2	0	3.08	37.58	**12.50**	1.00E+06	8.00E+04

Supplementary Figures

Figure S1:

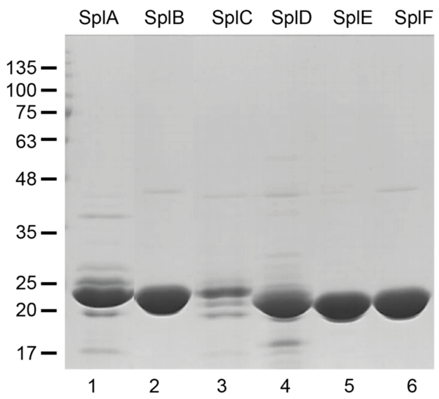

Figure S2:

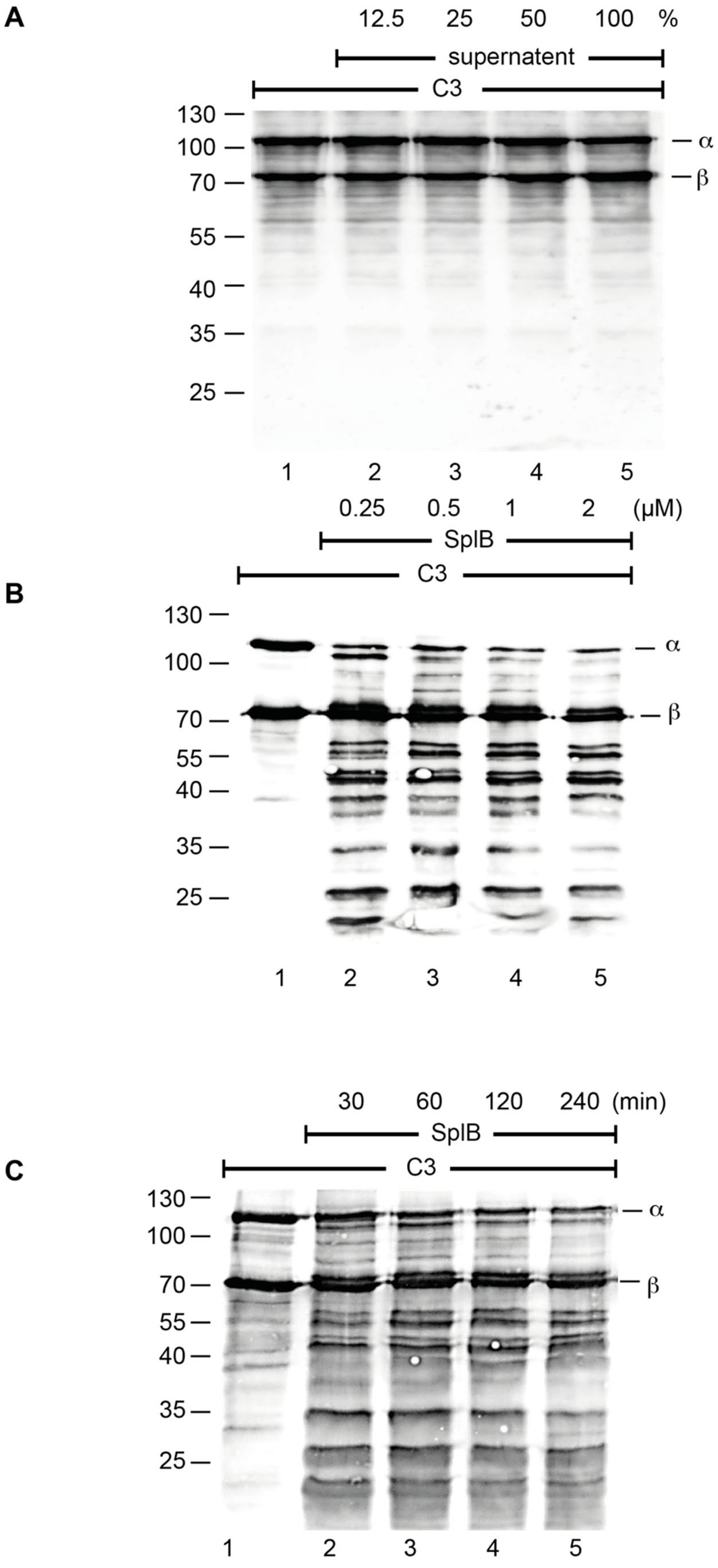

A
12.5 25 50 100 %
supernatent
C3
130
100
70
55
40
35
25
α
β
1 2 3 4 5
B
0.25 0.5 1 2 (µM)
SplB
C3
130
100
70
55
40
35
25
α
β
1 2 3 4 5
C
30 60 120 240 (min)
SplB
C3
130
100
70
55
40
35
25
α
β
1 2 3 4 5

Figure S3

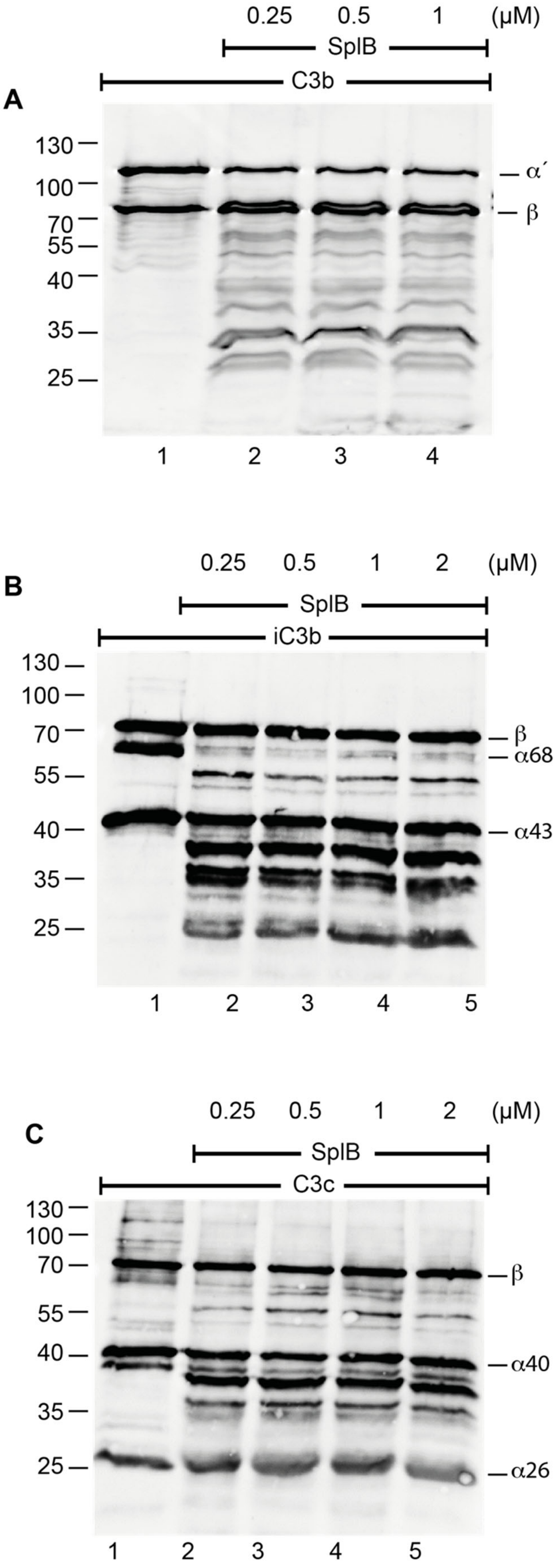
A
0.25 0.5 1 (μM)
SplB
C3b
130
100
70
55
40
35
25
α´
β
1 2 3 4
B
0.25 0.5 1 2 (μM)
SplB
iC3b
130
100
70
55
40
35
25
β
α68
α43
1 2 3 4 5
C
0.25 0.5 1 2 (μM)
SplB
C3c
130
100
70
55
40
35
25
β
α40
α26
1 2 3 4 5

Figure S4

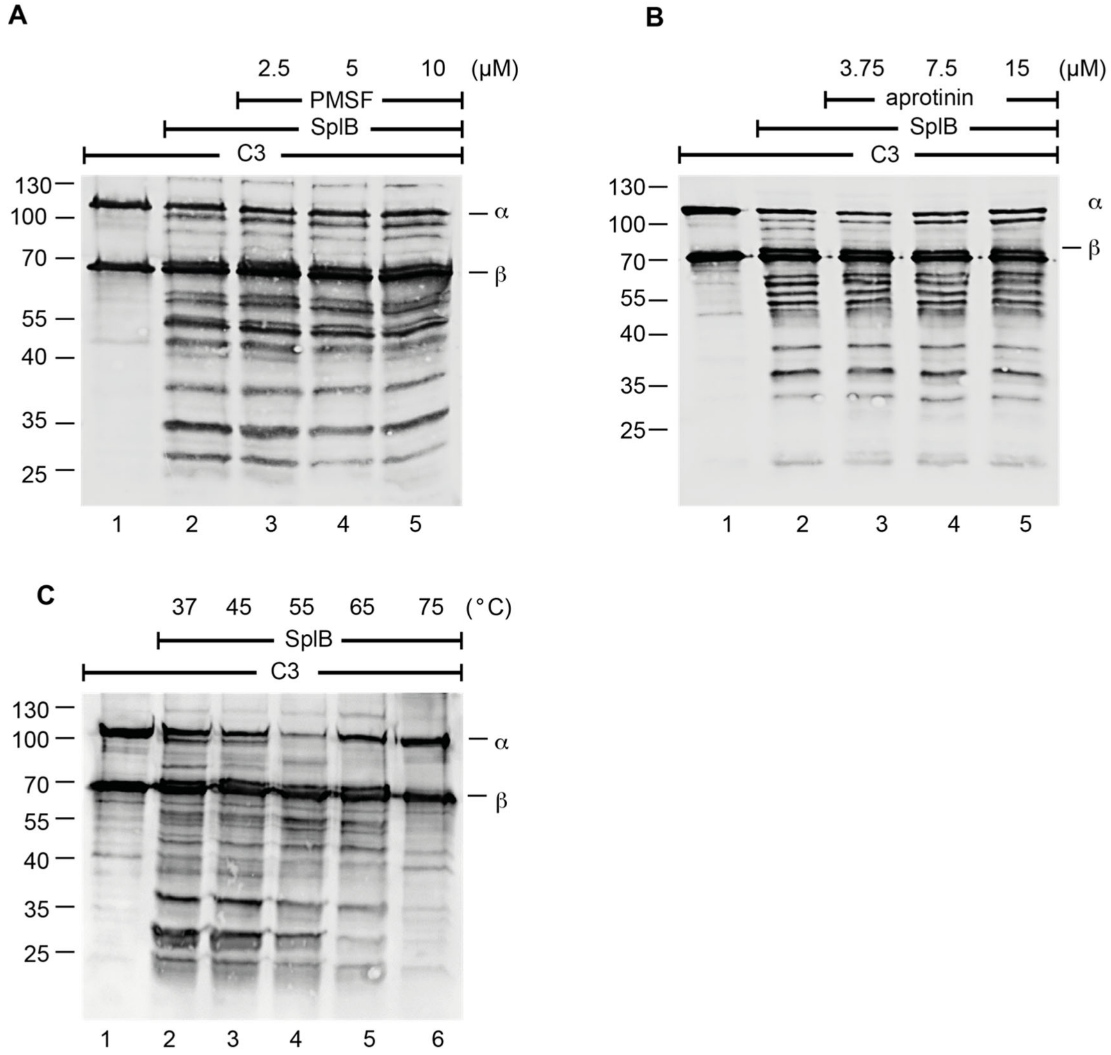
A
2.5
5
10
(μM)
PMSF
SplB
C3
130
100
70
55
40
35
25
α
β
1
2
3
4
5
B
3.75
7.5
15
(μM)
aprotinin
SplB
C3
130
100
70
55
40
35
25
α
β
1
2
3
4
5
C
37
45
55
65
75
(°C)
SplB
C3
130
100
70
55
40
35
25
α
β
1
2
3
4
5
6

Figure S5

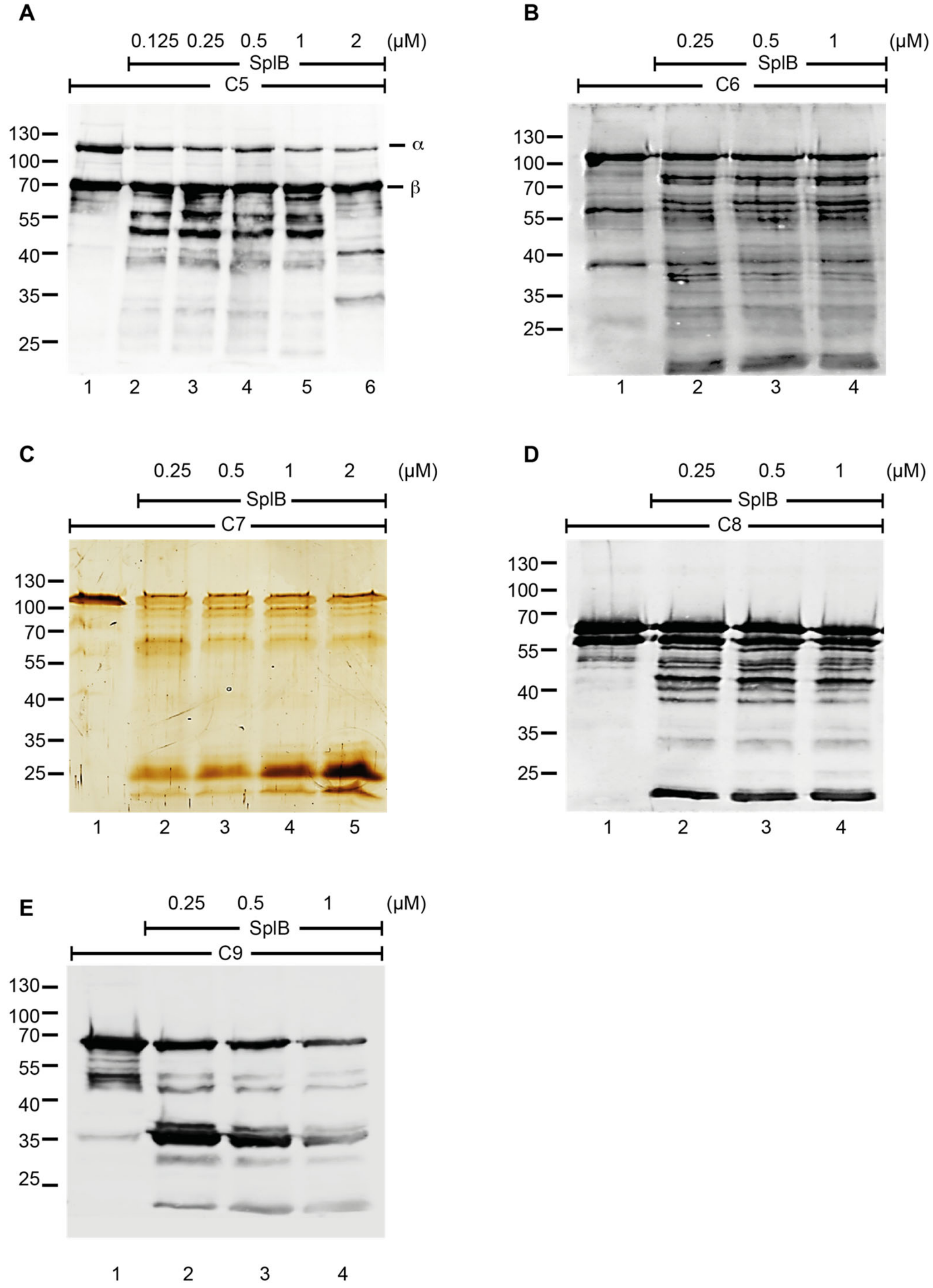
A
0.125 0.25 0.5 1 2 (μM)
SplB
C5
130
100
70
55
40
35
25
α
β
1 2 3 4 5 6
B
0.25 0.5 1 (μM)
SplB
C6
130
100
70
55
40
35
25
1 2 3 4
C
0.25 0.5 1 2 (μM)
SplB
C7
130
100
70
55
40
35
25
1 2 3 4 5
D
0.25 0.5 1 (μM)
SplB
C8
130
100
70
55
40
35
25
1 2 3 4
E
0.25 0.5 1 (μM)
SplB
C9
130
100
70
55
40
35
25
1 2 3 4

Figure S6

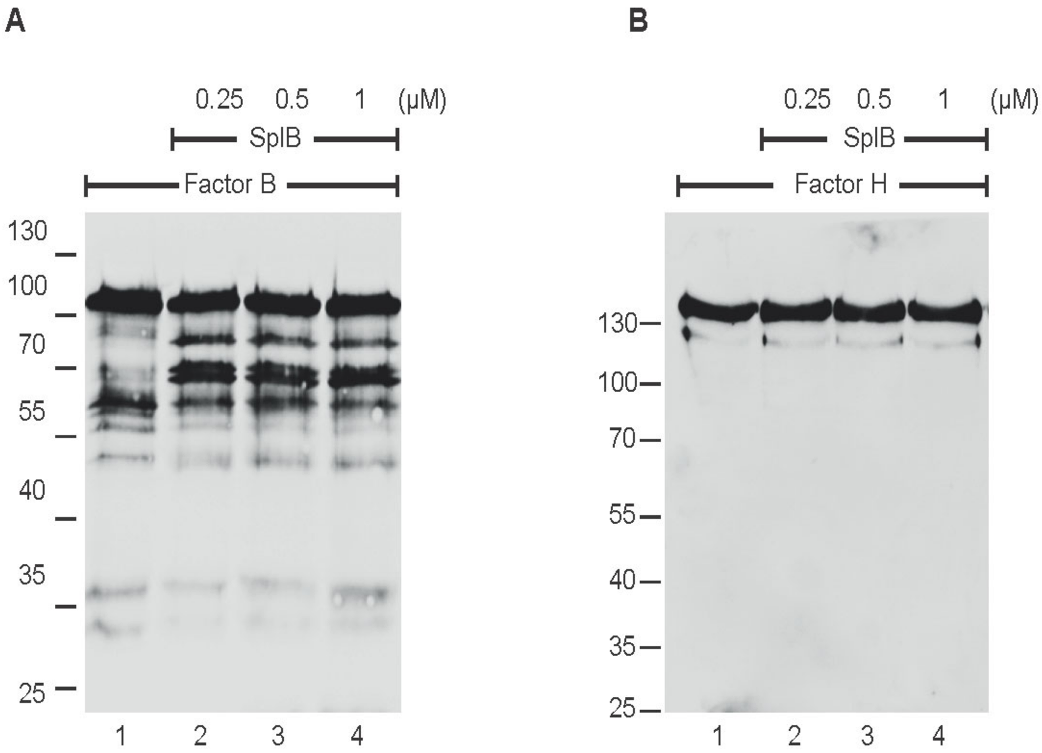
A
0.25 0.5 1 (μM)
SplB
Factor B
130
100
70
55
40
35
25
1 2 3 4
B
0.25 0.5 1 (μM)
SplB
Factor H
130
100
70
55
40
35
25
1 2 3 4

Figure S7

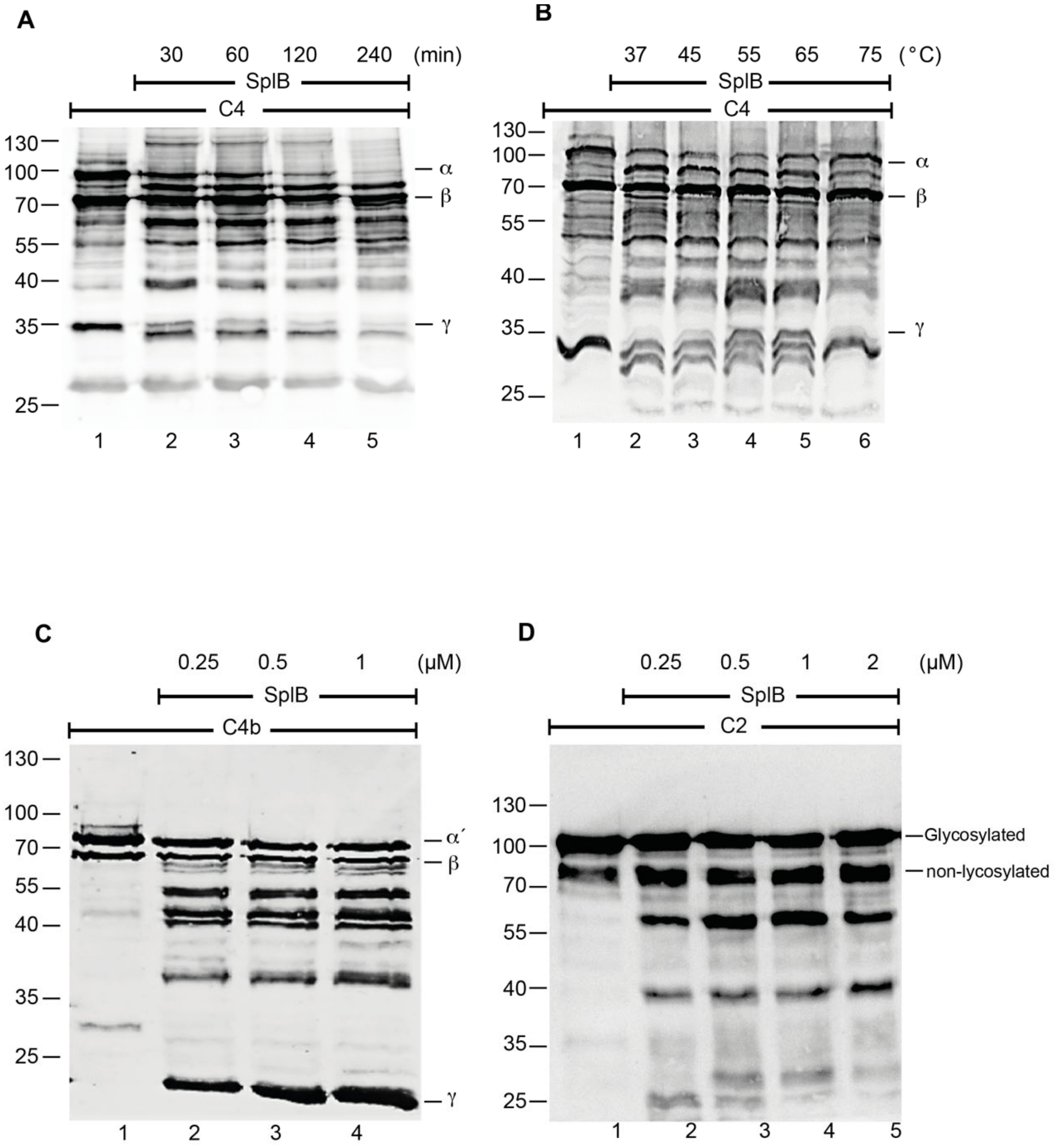
A
30 60 120 240 (min)
SplB
C4
130
100
70
55
40
35
25
α
β
γ
1 2 3 4 5
B
37 45 55 65 75 (°C)
SplB
C4
130
100
70
55
40
35
25
α
β
γ
1 2 3 4 5 6
C
0.25 0.5 1 (μM)
SplB
C4b
130
100
70
55
40
35
25
α´
β
γ
1 2 3 4
D
0.25 0.5 1 2 (μM)
SplB
C2
130
100
70
55
40
35
25
Glycosylated
non-lycosylated
1 2 3 4 5

Figure S8

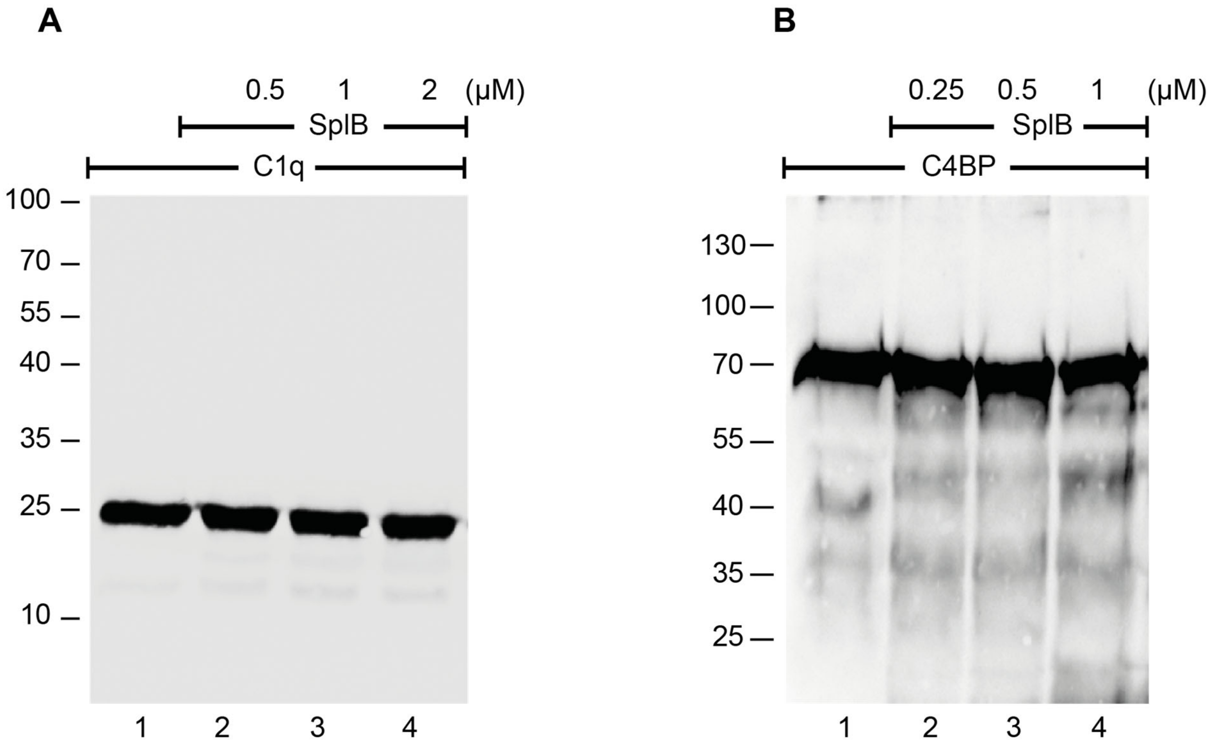
A
0.5
1
2
(µM)
SplB
C1q
100
70
55
40
35
25
10
1
2
3
4
B
0.25
0.5
1
(µM)
SplB
C4BP
130
100
70
55
40
35
25
1
2
3
4

Figure S9

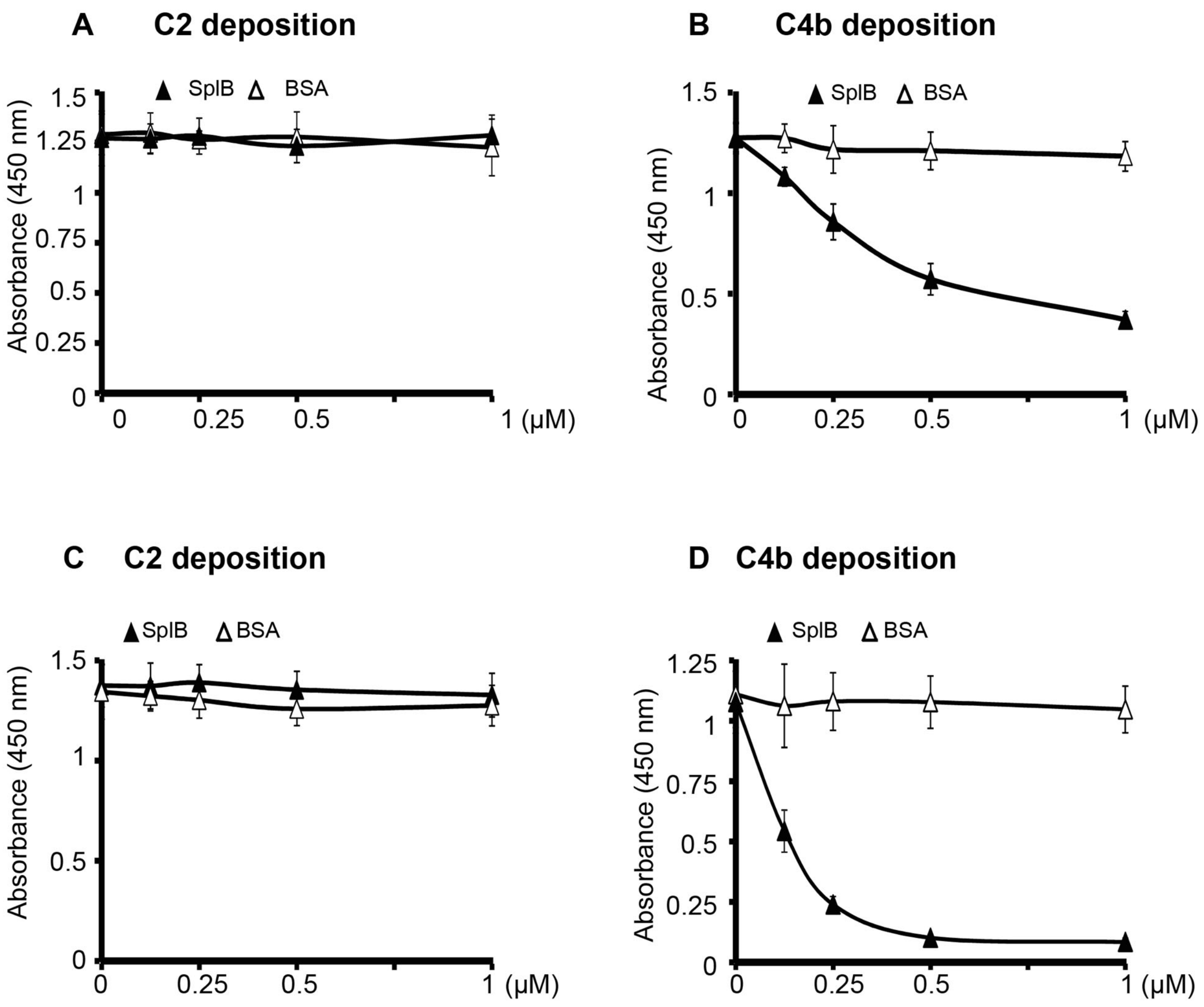
A C2 deposition
▲ SplB △ BSA
Absorbance (450 nm)
1.5
1.25
1
0.75
0.5
0.25
0
0
0.25
0.5
1 (µM)
B C4b deposition
▲ SplB △ BSA
Absorbance (450 nm)
1.5
1
0.5
0
0
0.25
0.5
1 (µM)
C C2 deposition
▲SplB △BSA
Absorbance (450 nm)
1.5
1
0.5
0
0
0.25
0.5
1 (µM)
D C4b deposition
▲ SplB △BSA
Absorbance (450 nm)
1.25
1
0.75
0.5
0.25
0
0
0.25
0.5
1 (µM)

Figure S10

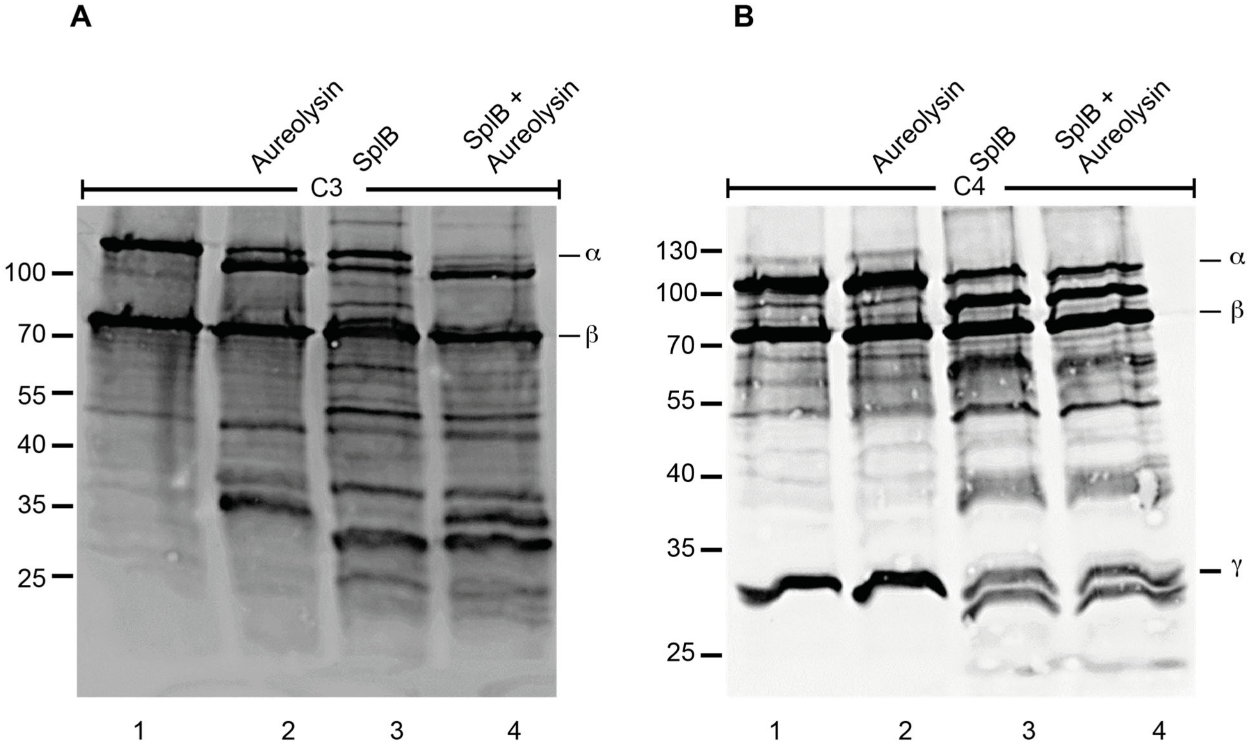
A
Aureolysin
SplB
SplB + Aureolysin
C3
100
70
55
40
35
25
α
β
1
2
3
4
B
Aureolysin
SplB
SplB + Aureolysin
C4
130
100
70
55
40
35
25
α
β
γ
1
2
3
4

Legend for Supplementary Figures

Figure S1. Recombinant Spl proteins

Preparations of the tag-free recombinant proteins SplA-SplF that were expressed in a protease-deficient *B. subtilis* strain and purified as native proteins. The proteins were analyzed by SDS-PAGE followed by staining with Coomassie-Blue.

Figure S2. Cleavage of the complement protein C3 by SplB

(A) *S. subtilis* without Spl-expression vector did not release proteases able to target C3; both the α- and the β-chains of C3 remained intact. Bacterial culture supernatant from *B. subtilis* 6051HGW LS8P-D without Spl exression vector was incubated with C3 at the indicated concentrations for 2 h at 37 °C. **(B/C)** Recombinant SplB was added to purified C3 and cleavage activity was evaluated by Western blotting with goat anti-human C3 antiserum. (**B**) SplB was incubated with purified C3 for 2 h at 37 °C at the indicated concentrations. The α- but not the β-chain of C3 was cleaved dependent on the SplB concentration. (**C**) SplB (1 µM) was incubated with purified C3 for the indicated time periods. Cleavage was time dependent.

Figure S3. Cleavage of complement component C3 activation fragments by SplB

Recombinant SplB was added to the C3 activation fragments C3b (**A**), iC3b (**B**) or C3d (**C**) at the indicated concentrations for 2 h at 37 °C. The cleavage activity was evaluated by western blotting with goat anti-human C3 antiserum. The protease cleaved predominantly the α-chains of these fragments.

Figure S4. Stability of the protease SplB

Recombinant SplB expressed by *B. subtilis* (1 µM) was incubated with purified complement proteins C3 for 1 h and the cleavage patterns of the complement proteins were analyzed by western blotting using appropriate antisera. Neither the serine protease inhibitor (**A**) PMSF (2.5-10 mM) nor (**B**) aprotinin (3.75.15 mM) inhibited C3 cleavage by SplB. (**C**) To examine the thermostability of SplB, the recombinant protease was heat-treated for 1 h at the indicated temperatures before incubation with purified C3. SplB was remarkably thermostable. Significant loss of enzymatic activity occurred only from 65 °C.

Figure S5. SplB cleaves all complement components of terminal complement forming the membrane attack complex (MAC).

Recombinant SplB was incubated with the purified complement components C5 (**A**), C6 (**B**), C7 (**C**), C8 (**D**) or C9 (**E**) at the indicated concentrations for 1 h at 37 °C, and cleavage patterns were visualized using western blotting with appropriate antisera, except for C7, where silver staining was used. SplB cleaved all tested complement proteins in a concentration-dependent fashion.

Figure S6. SplB cleaves Factor B, but not Factor H

SplB was incubated with purified factor B and factor H and the cleavage products were analyzed by western blotting. SplB cleaved factor B of the alternative complement pathway dose dependently (**A**) but spared the complement regulator factor H under the same conditions (**B**).

Figure S7. Proteolytic activity of SplB on initiating components of the classical and lectin pathways of complement

SplB was incubated with purified complement proteins and then the mixtures were separated by SDS-PAGE. Cleavage of complement proteins were analyzed with the appropriate goat anti-human antisera by western blotting. SplB (1 μM) cleaved the α-chain of C4 in a time-dependent manner (**A**). The enzymatic activity of the protease SplB was exquisitely heat stable (**B**). SplB further cleaved C4b (**C**) and C2 (**D**).

Figure S8. N-terminal peptides of the C4 fragments generated by SplB

Amino acid sequence of C4 with color coding of the α-chain (blue), the β-chain (green), and the γ-chain (orange). The N-terminal peptides generated by SplB-mediated cleavage of the C4 β-chain were determined with TAILS and are shown in bold letters. Proposed cleavage sites are depicted as red bars.

Figure S9. **SplB inhibits surface deposition of C4b, but not of C2**.

To examine the effect of SplB on the classical pathway and the lectin pathway, NHS (2%) served as the source of complement and was pre-incubated with SplB or BSA at the indicated concentrations for 1 h at 37 °C. The reaction mixtures were added to microtiter plates pre-coated with IgM to assess the classical pathway (**A, B**) or with mannan to analyze the lectin pathway (**C, D**) and incubated for 20 min at 37 °C. Deposition of the complement components C2 (**A, C**) and C4b (**B, D**) was measured**.** SplB but not the control protein BSA inhibited the deposition of C4b on the pre-coated

microtiter plates, while C2 deposition remained unaffected. Thus, SplB inhibited both the classical pathway and the lectin pathway at the level of C4.

Figure S10. SplB synergizes with aureolysin in cleavage of C3.

Aureolysin (1 µM), SplB (1 µM), or aureolysin together with SplB (1 µM each) were incubated with purified C3 (**A**) or C4 (**B**), and the cleavage was analyzed by Western blotting using appropriate antisera. (**A**) Both *S. aureus* proteases synergistically targeted primarily the α-chain of C3. The aureolysin-generated fragment C3bL was further degraded by SplB. (**B**) Aureolysin did not cleave C4, nor did aureolysin influence SplB mediated C4 cleavage.
